# *S*-acylated and nucleus-localized SALT OVERLY SENSITIVE3/CALCINEURIN B-LIKE4 stabilizes GIGANTEA to regulate Arabidopsis flowering time under salt stress

**DOI:** 10.1093/plcell/koac289

**Published:** 2022-09-22

**Authors:** Hee Jin Park, Francisco M Gámez-Arjona, Marika Lindahl, Rashid Aman, Irene Villalta, Joon-Yung Cha, Raul Carranco, Chae Jin Lim, Elena García, Ray A Bressan, Sang Yeol Lee, Federico Valverde, Clara Sánchez-Rodríguez, Jose M Pardo, Woe-Yeon Kim, Francisco J Quintero, Dae-Jin Yun

**Affiliations:** Department of Biomedical Science and Engineering, Konkuk University, Seoul 05029, South Korea; Department of Biological Sciences, Chonnam National University, Gwangju 61186, South Korea; Institute of Plant Biochemistry and Photosynthesis, Consejo Superior de Investigaciones Cientificas and Universidad de Sevilla, Seville 41092, Spain; Department of Biology, ETH Zurich, Zurich 8092, Switzerland; Institute of Plant Biochemistry and Photosynthesis, Consejo Superior de Investigaciones Cientificas and Universidad de Sevilla, Seville 41092, Spain; Division of Applied Life Science (BK21plus Program), Research Institute of Life Sciences, Plant Molecular Biology and Biotechnology Research Center, Graduate School of Gyeongsang National University, Jinju 52828, South Korea; Institut de Recherche sur la Biologie de l’Insecte, Université de Tours, 37200 Tours, France; Division of Applied Life Science (BK21plus Program), Research Institute of Life Sciences, Plant Molecular Biology and Biotechnology Research Center, Graduate School of Gyeongsang National University, Jinju 52828, South Korea; Institute of Plant Biochemistry and Photosynthesis, Consejo Superior de Investigaciones Cientificas and Universidad de Sevilla, Seville 41092, Spain; Department of Biomedical Science and Engineering, Konkuk University, Seoul 05029, South Korea; Institute of Plant Biochemistry and Photosynthesis, Consejo Superior de Investigaciones Cientificas and Universidad de Sevilla, Seville 41092, Spain; Department of Horticulture and Landscape Architecture, Purdue University, West Lafayette, Indiana 47907, USA; Division of Applied Life Science (BK21plus Program), Research Institute of Life Sciences, Plant Molecular Biology and Biotechnology Research Center, Graduate School of Gyeongsang National University, Jinju 52828, South Korea; Institute of Plant Biochemistry and Photosynthesis, Consejo Superior de Investigaciones Cientificas and Universidad de Sevilla, Seville 41092, Spain; Department of Biology, ETH Zurich, Zurich 8092, Switzerland; Institute of Plant Biochemistry and Photosynthesis, Consejo Superior de Investigaciones Cientificas and Universidad de Sevilla, Seville 41092, Spain; Division of Applied Life Science (BK21plus Program), Research Institute of Life Sciences, Plant Molecular Biology and Biotechnology Research Center, Graduate School of Gyeongsang National University, Jinju 52828, South Korea; Institute of Agriculture & Life Sciences, Graduate School of Gyeongsang National University, Jinju 52828, South Korea; Institute of Plant Biochemistry and Photosynthesis, Consejo Superior de Investigaciones Cientificas and Universidad de Sevilla, Seville 41092, Spain; Department of Biomedical Science and Engineering, Konkuk University, Seoul 05029, South Korea; Key Laboratory of Molecular Epigenetics of the Ministry of Education (MOE), Northeast Normal University, Changchun 130024, China

## Abstract

The precise timing of flowering in adverse environments is critical for plants to secure reproductive success. We report a mechanism in Arabidopsis (*Arabidopsis thaliana*) controlling the time of flowering by which the *S*-acylation-dependent nuclear import of the protein SALT OVERLY SENSITIVE3/CALCINEURIN B-LIKE4 (SOS3/CBL4), a Ca^2+^-signaling intermediary in the plant response to salinity, results in the selective stabilization of the flowering time regulator GIGANTEA inside the nucleus under salt stress, while degradation of GIGANTEA in the cytosol releases the protein kinase SOS2 to achieve salt tolerance. *S*-acylation of SOS3 was critical for its nuclear localization and the promotion of flowering, but partly dispensable for salt tolerance. SOS3 interacted with the photoperiodic flowering components GIGANTEA and FLAVIN-BINDING, KELCH REPEAT, F-BOX1 and participated in the transcriptional complex that regulates *CONSTANS* to sustain the transcription of *CO* and *FLOWERING LOCUS T* under salinity. Thus, the SOS3 protein acts as a Ca^2+^- and *S*-acylation-dependent versatile regulator that fine-tunes flowering time in a saline environment through the shared spatial separation and selective stabilization of GIGANTEA, thereby connecting two signaling networks to co-regulate the stress response and the time of flowering.

## Teaser

Teaser Abstract

The SALT OVERLY SENSITIVE3/CALCINEURIN B-LIKE4 protein acts as a Ca^2+^- and *S*-acylation-dependent regulator that fine-tunes flowering time in a saline environment through the selective stabilization of GIGANTEA in the nucleus.

IN A NUTSHELL **Background:** For plants, extremes in the cardinal conditions of light, temperature, nutrients, and water availability are major drivers of natural selection. Adaptive responses must be coupled to adjustments in the reproductive strategy to be favored by selection. In unfavorable environments, the transition to flowering is adjusted earlier or later to maximize the production of dormant structures (seeds) that can survive prolonged adverse episodes and eventually reinitiate a life cycle when conditions improve. Water and nutrient deprivation can quickly compromise survival and generally induce earlier flowering, whereas soil salinity reduces growth and delays flowering.

**Question:** What are the molecular mechanisms by which the plant *Arabidopsis thaliana* readjusts flowering time in response to salinity stress to secure reproductive success?

**Findings:** The protein GIGANTEA (GI) synchronizes flowering time with the photoperiodic and circadian rhythms. Salinity results in rapid degradation of GI protein and delayed flowering. We show that GI is selectively degraded in the cytoplasm but protected inside the nucleus by a mechanism that involves the addition of a fatty acid (palmitoylation) and nuclear import of the calcium-signaling protein SALT OVERLY SENSITIVE3/CALCINEURIN B-LIKE4 (SOS3/CBL4). Moreover, nuclear SOS3/CBL4 participates in the transcriptional complex that regulates *CONSTANS*, a key gene governing flowering time, thereby creating a molecular switch that integrates calcium signaling, spatial segregation of proteins, and transcriptional regulation to adjust the time of flowering in a saline environment.

**Next steps:** The timing of flowering and the environmental resilience of crops are critical traits in agriculture. Understanding how stress responses dictate when plants flower, and produce their fruits and seeds, will be critical for food security under the threat of climate change.

## Introduction

Natural selection of different biological forms and functions occurs in variable physical environments. Depending on the specific environment, different traits are favored for the reproduction and perpetual survival of the species. For plants, extremes in the cardinal conditions such as light, temperature, and, most importantly, the quantity and quality of available water and nutrients are among the major drivers of natural selection (Maggio et al., 2018). Adaptive responses must be coupled to adjustments in the reproductive strategy to be favored by selection. Seasonal changes, especially in temperature and day length, provide key signals setting the time of flowering. However, depending on the dynamics of environmental stressors, transition to flowering is adjusted earlier or later to maximize the production of dormant structures (seeds) that can survive prolonged adverse episodes and eventually reinitiate a life cycle (Kazan and Lyons, 2016). Environmental stressors such as water and nutrient deprivation generally induce earlier flowering (Kazan and Lyons, 2016; Takeno, 2016), whereas salinity delays flowering (Kim et al., 2007b, 2013a; Li et al., 2007; Ryu et al., 2014).

In the model plant Arabidopsis (*Arabidopsis thaliana*), the major signaling systems that perceive environmental cues and initiate flowering converge on a few key integrators. *CONSTANS* (*CO*) is a central hub of the photoperiodic flowering pathway that activates the expression of the floral inductor (florigen) *FLOWERING LOCUS T* (*FT*) in long-day (LD) conditions (Suarez-Lopez et al., 2001; Turck et al., 2008). CO accumulation is strictly regulated both at the transcriptional and posttranslational levels to ensure that its activity coincides with the lengthening of the days and to optimize seasonal flowering. *CO* diurnal expression is regulated by the opposing action of activators and repressors controlled by the circadian clock, including GIGANTEA (GI), FLOWERING BHLHs (FBHs), and CYCLING DOF FACTORs (CDF1, CDF2, CDF3, and CDF5) that promote the presence of *CO* mRNA in the evenings in LD but not in short-day (SD) conditions (Kinoshita and Richter, 2020). The abundance of CDF proteins is in turn depressed by the blue light receptor F-box E3 ubiquitin ligase FLAVIN-BINDING, KELCH REPEAT, F-BOX1 (FKF1; Imaizumi et al., 2005; Fornara et al., 2009). The clock protein GI interacts with and stabilizes FKF1 in a blue light-dependent manner, promoting the degradation of CDF proteins and *CO* expression in LD conditions (Sawa et al., 2007). GI can also neutralize *FT* repressors to enable *FT* transcription and promote transition to flowering (Sawa and Kay, 2011). On the other hand, CO protein stability is diurnally regulated by photoreceptors and the proteasome (Valverde et al., 2004; Imaizumi et al., 2005; Sawa et al., 2007; Fornara et al., 2009). This way, red light sensed by phytochrome B (PHYB) induces its degradation in the morning by the HIGH EXPRESSION OF OSMOTICALLY RESPONSIVE GENES1 (HOS1) E3 ubiquitin ligase while the E3 ligase CONSTITUTIVE PHOTOMORPHOGENESIS1 (COP1) promotes its degradation at night (Jang et al., 2008; Lazaro et al., 2012). Blue and far-red lights are sensed by CHRYPTOCHROME2 (CRY2) and phytochrome A (PHYA) to promote CO stabilization in the evening by repressing COP1 function, a process in which FKF1 and the PSEUDO RESPONSE REGULATORS (PRRs) are also involved (Kinoshita and Richter, 2020).

Salt stress delays flowering time in Arabidopsis by repressing expression of *CO* and *FT* (Kim et al., 2007b; Li et al., 2007; Ryu et al., 2014). In parallel, salinity promotes extension of vegetative growth by stabilizing DELLA proteins that act as repressors of cell proliferation, expansion, and flowering (Achard et al., 2006). Other regulators mediating abiotic stress responses are also known to modulate flowering time and vice versa, but mechanistic insights are still largely missing (Park et al., 2016). Among these dual effectors is GI, which has emerged as a central hub coordinating the photoperiodic flowering pathway and stress responses against drought, cold, salt, light, and carbohydrate metabolism (Park et al., 2016). The involvement of GI in stress responses includes transcriptional regulation of downstream genes (Fornara et al., 2015) and the interaction with circadian and other signaling components that in turn affect various physiological adaptations (Greenham and McClung, 2015; Seo and Mas, 2015).

GI influences salinity tolerance in Arabidopsis through the direct association with key signaling components of the salinity stress response (Kim et al., 2013a). In high salinity, plants utilize the SALT OVERLY SENSITIVE (SOS) pathway to maintain ion homeostasis (Ji et al., 2013). The core components of the SOS pathway comprise the Na^+^/H^+^ antiporter SOS1, the Ser/Thr protein kinase SOS2/CIPK24, and two alternative calcium binding proteins, SOS3/CBL4 and SCaBP8/CBL10, that activate and recruit SOS2 to cellular membranes (Qiu et al., 2002; Quintero et al., 2002; Kim et al., 2007a; Quan et al., 2007; Quintero et al., 2011). SOS2 activates Na^+^ efflux by phosphorylating SOS1 at its C-terminal autoinhibitory domain (Quintero et al., 2011). In regular growth conditions, GI makes a complex with and inhibits SOS2 (Kim et al., 2013a). Salt stress causes the degradation of GI protein by the 26S proteasome and the release of SOS2, which is then free to interact with SOS3, activate SOS1 and mount the adaptation to the saline environment. The removal of GI leads to exceptional salt tolerance at least in part by mimicking Na^+^-induced GI degradation, whereas plants overexpressing *GI* exhibit a salt-sensitive phenotype by sequestering SOS2. The precise mechanism triggering the dissociation of the GI–SOS2 complex and GI degradation under salt stress has not been resolved, although indirect evidence suggested that the Ca^2+^-sensor protein SOS3 played a role since excess SOS3 interfered with GI-SOS2 complex formation (Kim et al., 2013a). Moreover, SOS3 has been reported to have an indeterminate role in influencing flowering time as the *sos3-1* mutant, which has impaired calcium binding, showed late flowering under salt stress (Li et al., 2007). The molecular basis of this phenotype has remained unexplained.

We have addressed the molecular mechanism by which SOS3 helps resetting the flowering time under salt stress. We show here that SOS3 acts as a crucial regulator of flowering under saline stress through a mechanism that involves the nuclear entry of SOS3 and the stabilization of GI specifically inside the nucleus. Under regular growth conditions, GI partitions between the nucleoplasm and cytoplasm (Kim et al., 2013b). Upon salinity stress, only cytoplasmic GI is degraded, thereby releasing SOS2 to mount the salt stress response, whereas nuclear GI remains stable in physical association with SOS3, eventually leading to flowering. Nuclear import of SOS3 requires *S*-acylation (palmitoylation). We further demonstrate that SOS3 interacts with GI and FKF1, and participates in the transcriptional complex that promotes transcription of *CO*. These results reveal the intimate molecular linkages of networks controlling salinity stress responses and the adaptive initiation of flowering under adverse environments. They also reveal a novel mechanism for transcriptional regulation of flowering determinants by a Ca^2+^-activated stress protein whose nuclear entry is controlled by *S*-acylation.

## Results

### SOS3 controls flowering under saline stress through the CO-FT pathway

**Figure 1:**
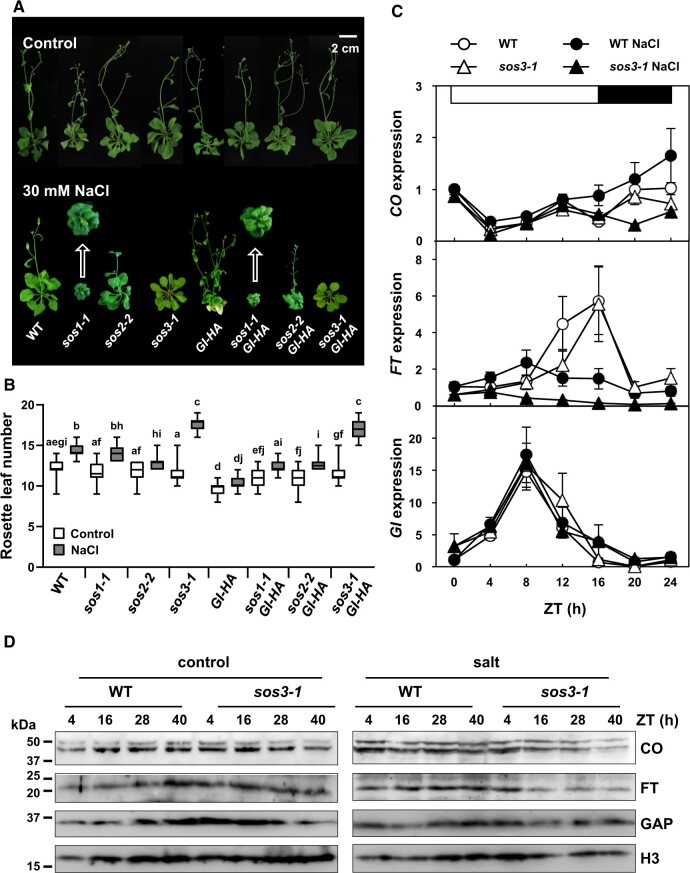
SOS3 controls flowering under salt stress through the CO/FT pathway. A, Effect of salt on the flowering time in wild-type Col-0 *gl1* (WT), and mutants *sos1-1*, *sos2-2*, and *sos3-1*, either overexpressing or not overexpressing *GIGANTEA (GI-HA*). Eight-day-old seedlings were transferred to MS media with or without 30 mM NaCl. The photographs were taken after bolting. Representative plants are shown. B, Rosette leaf number at bolting time of plants grown with and without salt, as in (A), to score flowering time. Data are shown as box plots: center lines show the medians; box limits indicate the 25th and 75th percentiles; whiskers extend to the minimum and maximum (*n* = 16 plants). Letters indicate statistical differences at *P* < 0.01, Fisher’s LSD test; means with the same letter are statistically similar. C, Transcript levels of *CO*, *FT* and *GI* in WT (Col-0 *gl1*) and *sos3-1* mutant plants. Two-week-old plants grown in LDs were left untreated (open symbols) or treated with 100 mM NaCl (filled symbols) at the beginning of the light period (ZT0) and harvested every 4 h. Total RNA was isolated and transcript levels of *CO*, *FT*, and *GI* were measured by RT-qPCR and normalized to that of *At5g12240* by the comparative cycle threshold (ΔΔCt) method. Data are means ± SEM from three biological replicates (plants) with three technical repeats each. A complete statistical analysis of data variance is included in Supplemental Data Set S1. The white and black bars on top indicate light and dark periods. D, CO and FT protein levels. Eight-day-old plants (Col-0 and *sos3-1* mutant) grown in LD were transferred to MS media with or without 100 mM NaCl at ZT1 (1 h after lights-on) and harvested at ZT4 and ZT16 for two consecutive days. Total protein was extracted and probed with rabbit α-CO and α-FT antibodies. Glyceraldehyde-3-phosphate dehydrogenase (GAP) and Histone3 (H3) were used as loading controls. Protein size markers are on the left. The experiment was repeated twice with similar results.

Previously, we have shown that GI, which promotes photoperiodic-dependent flowering in LDs, also functions to restrain the activity of the SOS pathway by sequestering SOS2 (Kim et al., 2013a). Upon salt stress, the GI-SOS2 complex dissociates and GI degrades to delay flowering. Under regular growth conditions, *sos1-1*, *sos2-2*, and *sos3-1* mutants flowered as did the wild type (WT). Salt stress delayed flowering in the WT and *sos1-1* mutant, and to a lesser extent in the *sos2-2* mutant (Figure 1, A and B). Flowering of the *sos3-1* mutant was delayed further under salt stress compared to that of all other genotypes (Figure 1, A and B). The *sos1-1* mutant exhibited maximal sensitivity to 30 mM NaCl among the genotypes tested but still flowered normally, indicating that delayed flowering in *sos3-1* plants was not a direct consequence of Na^+^ toxicity.

Ultimately, salt stress delays flowering because of reduced transcript levels of the GI-regulated floral activator *FT* (Li et al., 2007; Kim et al., 2013a), whose expression in turn requires the transcription factor CO (Valverde et al., 2004). We found that salt stress altered the photoperiodic oscillation of *CO* transcripts and promoted an increase in *CO* transcripts throughout dusk and night (Figure 1C). *FT* transcript levels followed the opposite trend, with a marked decline after midday (ZT8) and losing the maxima at dusk (ZT16) typical of untreated controls (Suarez-Lopez et al., 2001). *CO* and *FT* transcript levels in the *sos3-1* mutant followed WT dynamics under control conditions, but salt treatment reduced *CO* transcript levels at night, thus departing from the WT behavior, and abated further the *FT* transcript levels compared to those of the WT (Figure 1C). By contrast, the diurnal dynamics of *GI* transcript levels were not affected by salt or the *sos3-1* mutation (Figure 1C). Salinity did not alter the transcript levels of other flowering genes such as *FKF1*, *SOC1*, *FLD* (*FLOWERING LOCUS D*), *FLC*, and *FCA* (*FLOWERING TIME CONTROL PROTEIN FCA*; Supplemental Figure S1).

Statistical analysis was performed with two-way ANOVA for time, genotype and treatment factors for *CO*, *FT*, and *GI* transcript levels (Supplemental Data Set S1). As expected from the diurnal oscillations in transcript levels of these genes, time of sampling (Zeitgeber Time, ZT) was the most significant source of variance. For *CO*, time was significant at *P *<* *0.0001 and genotype/treatment was significant at *P *<* *0.0001, with no significant interaction between them. For *FT*, time was significant at *P *<* *0.0001 and genotype/treatment was significant at *P *<* *0.0001, with a significant interaction between these factors at *P *<* *0.01. In *GI*, only time was significant, at *P *<* *0.0001; genotype/treatment had no significant effect. Immunoblotting confirmed a reduction in CO and FT protein levels at dusk in the *sos3-1* mutant under salt stress compared to those in the WT, with the decline starting in the evening of the first day of salt treatment (Figure 1D). Quantification of *CO* and *FT* transcript levels at Days 1 and 5 at two salinity levels (50 and 100 mM NaCl) showed that the salt- and genotype-dependent reduction in *CO* and *FT* expression was more intense after the 5-d salt treatment (Supplemental Figure S2). These results indicate that SOS3 not only mediates adaption to salinity through the SOS pathway but also participates in resetting flowering time under salt stress through the CO-FT module.

### SOS3 stabilizes GI in the nucleus under salt stress

Accumulation of the GI protein in the late afternoon of LDs promotes the transcription of floral activators *CO* and *FT* (Sawa et al., 2007; Sawa and Kay, 2011), and *GI* overexpression leads to early flowering (Kim et al., 2013b). Accordingly, overexpression of tagged *GI-HA* produced early flowering in otherwise WT plants both in control and saline conditions (Figure 1, A and B). The later flowering time of *sos1/GI* and *sos2/GI* plants relative to *GI-HA* plants under saline conditions is likely due to the greater intensity of the salt stress in the *sos* mutants, which would result in a faster rate of GI protein degradation compared to that in the WT/*GI* line (Kim et al., 2013a). Notably, GI-HA failed to suppress the late flowering of the *sos3-1* mutant under salt stress, which showed statistically the same flowering time regardless of *GI-HA* overexpression (Figure 1, A and B). Moreover, the severe flowering time delay of the *gi-1* mutant was not further aggravated in the *sos3-1 gi-1* double mutant (Supplemental Figure S3). Together, these results indicate that promotion of flowering requires a functional SOS3 protein under salinity stress but not under regular growth conditions, and that the effect of SOS3 on the transition to flowering is dependent on GI. However, the fact that salt treatment delayed further the flowering time of *gi-1* and *gi-1 sos3-1* plants indicated that other positive flowering regulators besides GI are likely affected by salinity stress.

**Figure 2:**
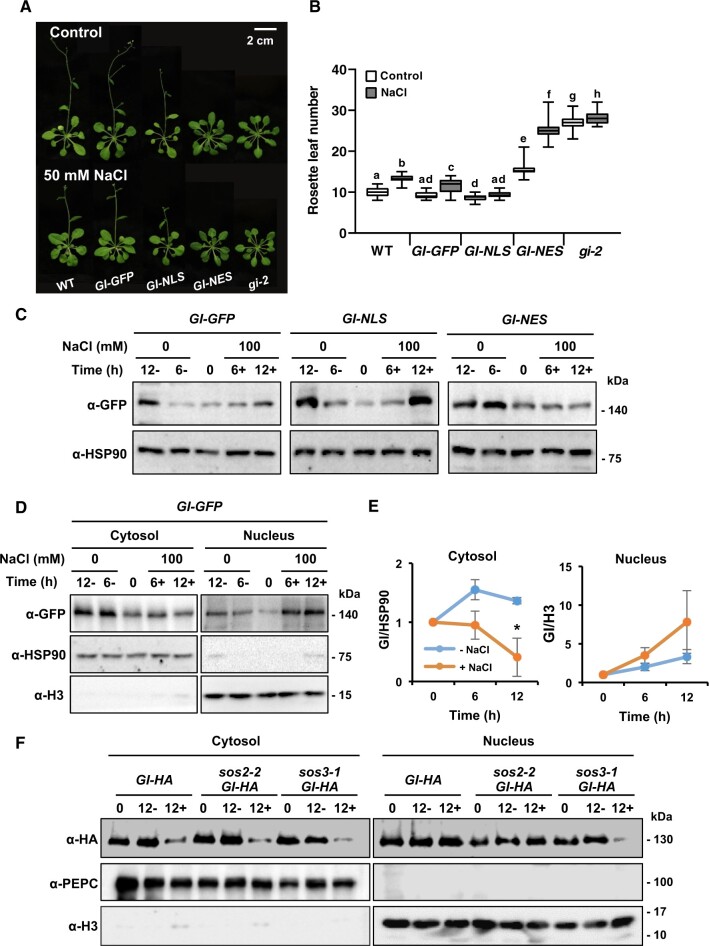
Stability of the nuclear fraction of GI controls time of flowering. A, Eight-day-old plants of wild-type Col-0 (WT) and the *gi-2* mutant transformed with *GI-GFP, GI-NLS and GI-NES* were grown in LD with or without 50 mM NaCl treatment. B, The rosette leaf number at bolting was used to determine flowering time. Data are shown as box plots: center lines are the medians; box limits indicate the 25th and 75th percentiles; whiskers extend to the minimum and maximum (*n* ≥ 15). Letters indicate significantly different means at *P* < 0.01, by Fisher’s LSD test. C, Total proteins were extracted from 2-week-old *GI-GFP*, *GI-NLS*, and *GI-NES* plants treated with or without 100 mM NaCl for 6 h and 12 h (indicated as 6+, 12+ and 6−, 12−, respectively). Immunoblots with α-GFP antibody were performed to detect GI protein in *GI-GFP*, *GI-NLS*, and *GI-NES* plants. α-HSP90 antibody was used as a loading control. D, Two-week-old *35S:GI-GFP* (*GI-GFP*) plants were treated with or without 100 mM NaCl at ZT0 and harvested at ZT6 and ZT12. Cytosolic and nuclear proteins were extracted and submitted to immunoblotting with α-GFP antibodies. α-HSP90 and α-H3 antibodies were used for cytosolic and nuclear markers, respectively. E, Quantification of GI protein abundance. Data represent means ± SEM from three independent biological replicates. Significant differences are indicated by asterisks (*P < 0.05 using a two-tailed Student’s *t* test). F, Cytosolic and nuclear proteins were extracted from 2-week-old *GI-HA*, *sos2-2 GI-HA* and *sos3-1 GI-HA* plants treated with (12+) or without (12−) 100 mM NaCl for 12 h. Immunoblots with α-HA antibody were performed to detect GI protein. α-PEPC and α-H3 antibodies were used for cytosolic and nuclear markers, respectively. This experiment was repeated 3 times with similar results.

GI is a nucleo-cytoplasmic protein, and forced spatial segregation of GI into nuclear or cytosolic compartments results in different flowering time outputs (Kim et al., 2013b). Transgenic plants exclusively expressing a recombinant GI protein fused to a nuclear localization signal (*proGI:GI-GFP-NLS* construct in the *gi-2* mutant, henceforth *GI-NLS*) showed early flowering compared to WT and transgenic plants expressing nucleo-cytoplasmic *proGI:GI-GFP* as a control (Figure 2, A and B). Conversely, transgenic plants expressing a GI protein fused to a nuclear export signal (*proGI:GI-GFP-NES* in *gi-2*, henceforth *GI-NES*) exhibited late flowering due to nuclear exclusion of GI. The late flowering of *GI-NES* plants was exacerbated under salt stress and resembled that of the untransformed *GI*-deficient mutant *gi-2* (Figure 2, A and B). These results are coherent with the finding that only the nuclear pool of GI was competent to promote photoperiodic flowering (Kim et al., 2013b) and suggest that the salt-induced delay of flowering resulting from a decrease in the steady-state levels of total GI protein (Kim et al., 2013a) affects primarily the cytosolic GI pool. Accordingly, both nucleo-cytoplasmic GI-GFP and nuclei-excluded GI-NES protein abundance declined upon salt stress whereas nucleus-localized GI-NLS resisted degradation (Figure 2C). Moreover, subcellular fractionation showed that salt stress diminished the cytosolic content of GI-GFP but not that of the nuclear localized protein (Figure 2, D and E). This result was confirmed by inspecting GFP fluorescence in salt-treated roots expressing GFP-tagged GI, and in whole seedlings of transgenic plants expressing a HA-tagged GI protein (Supplemental Figure S4). Collectively, these results indicate that preservation of GI stability inside the nucleus is critical to ensure flowering under salinity stress.

To examine whether SOS3 is involved in the salt-regulated GI stability, WT, *sos2-2* and *sos3-1* plants, all expressing *GI-HA*, were treated with 100 mM NaCl for 12 h starting at ZT2, and cytosolic and nuclear proteins were extracted (the *sos1-1* mutant was excluded from this assay owing to its extreme sensitivity to high-salt). Salt-induced degradation of cytosolic GI was found in all plant lines, whereas reduction of the nuclear GI pool was found only in *sos3-1 GI-HA* plants (Figure 2F). This result suggests that SOS3 is needed for the stabilization of the GI protein within the nucleus under salt stress. CBL10/SCaBP8 (CALCINEURIN B-LIKE10/SOS3-LIKE CALCIUM BINDING PROTEIN8), a homolog of SOS3/CBL4 that interacts with SOS2 to impart salt tolerance (Kim et al., 2007a; Quan et al., 2007), was not involved in the salinity-induced resetting of flowering time since enhanced flowering delay in salt was not observed in the *cbl10* mutant (Supplemental Figure S5).

### SOS3 interacts with GI in a calcium-dependent manner

**Figure 3:**
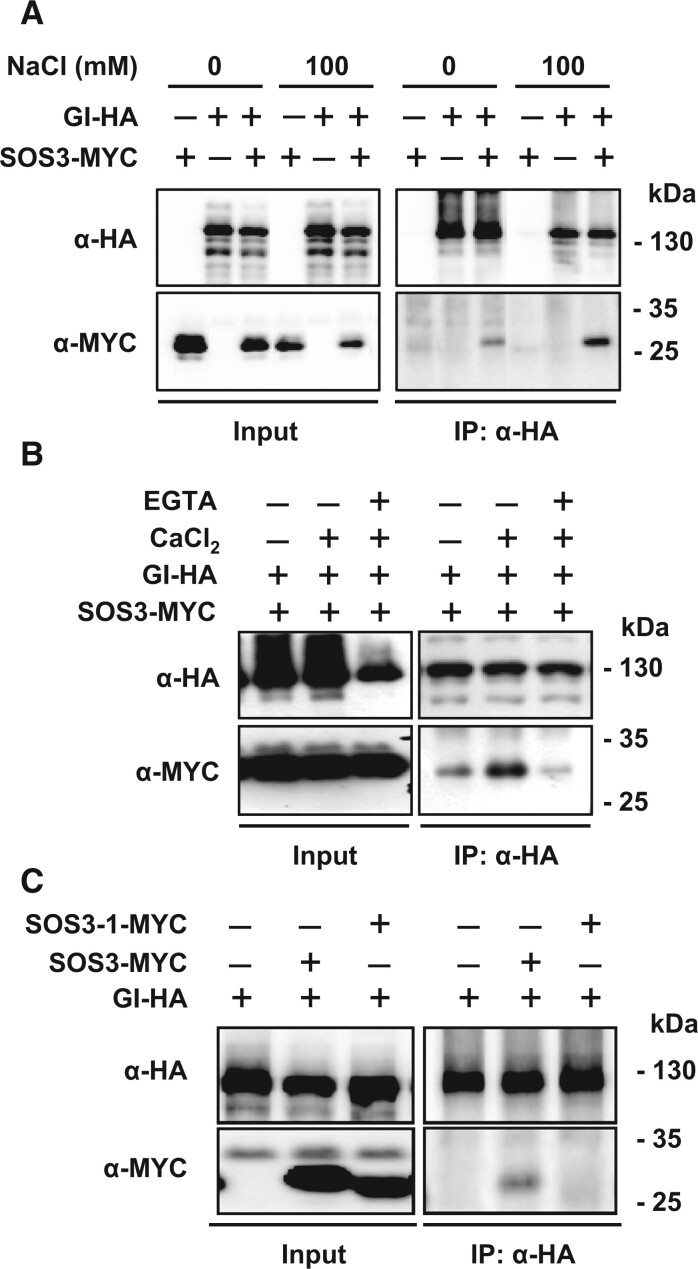
Salt- and Ca^2+^-dependent interaction of SOS3 with GI. A, Co-immunoprecipitation of SOS3 and GI. *Nicotiana benthamiana* leaves transiently expressing *SOS3-MYC* and *GI-HA* were treated with 100 mM NaCl for 8 h and total proteins were pulled down with HA antibodies (α-HA). The SOS3 protein was detected by MYC antibodies (α-MYC). B, Ca^2+^ effect on the interaction between SOS3 and GI. *Nicotiana benthamiana* leaves transiently expressing *SOS3-MYC* and *GI-HA* were treated with 3 mM CaCl_2_, or with 3 mM CaCl_2_ and 2 mM EGTA. C, The SOS3-1 protein with a mutated EF-hand motif cannot bind to GI.

Next, we tested SOS3 binding to GI and whether salinity influenced the interaction. Co-immunoprecipitation (co-IP) from *Nicotiana benthamiana* leaves showed that GI-HA interacted with SOS3-MYC (Figure 3). The interaction was enhanced by 100 mM NaCl and 3 mM Ca^2+^ treatments, whereas EGTA suppressed the interaction (Figure 3, A and B). Moreover, the mutant protein SOS3-1 bearing a three-amino acid deletion in the third Ca^2+^-binding EF-hand motif that abrogates interaction with SOS2 (Guo et al., 2004) failed to interact with GI (Figure 3C). The Ca^2+^-dependent interaction of SOS3 with GI was confirmed by BiFC in *N. benthamiana* (Supplemental Figure S6). Again, both NaCl and Ca^2+^ enhanced the interaction of GI and SOS3, EGTA repressed the interaction, and GI did not interact with the mutant protein SOS3-1.

### *S*-acylation of SOS3 is crucial for nuclear import and flowering under salt stress

**Figure 4:**
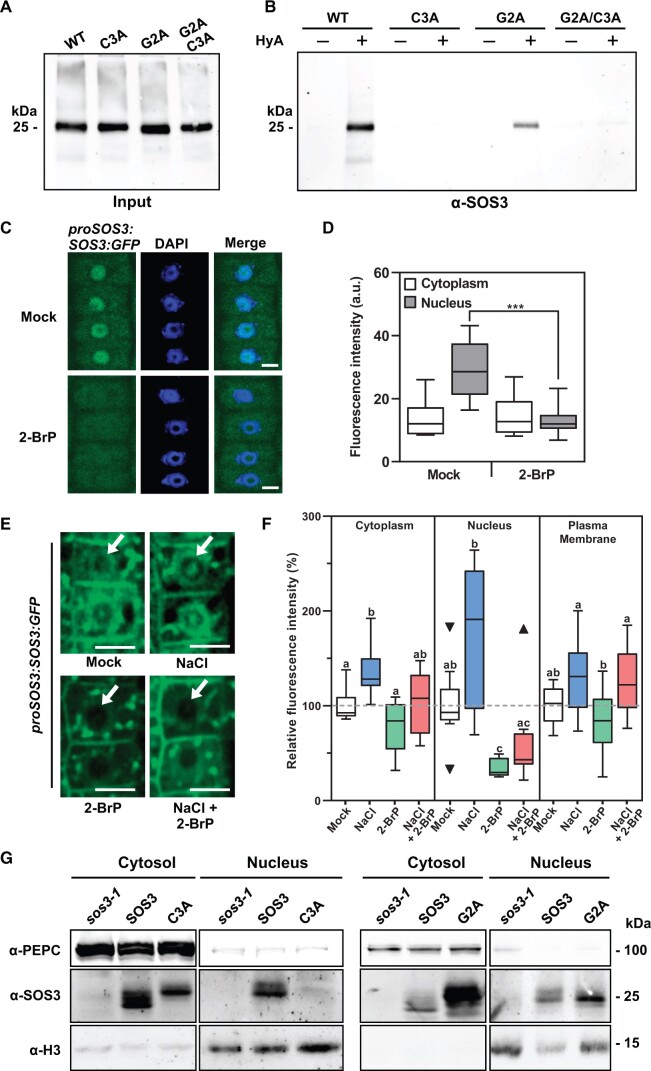
Palmitoylation directs SOS3 nuclear import. **A and B**, Wild-type SOS3 (WT), single mutants G2A and C3A, and the double mutant G2A/C3A, were expressed transiently in *N. benthamiana*. Leaf extracts were treated with 30 mM NEM under denaturing conditions to block free cysteine thiols, and proteins were acetone precipitated. Resuspended proteins were incubated with thiopropyl-sepharose 6B in the presence (+) or absence (–) of 0.5 M HyA to break palmitoyl thioester bonds. **A**, Control of total leaf proteins applied as input for acyl-RAC, probed with α-SOS3 antibodies. Each lane contains proteins corresponding to 0.5 mg leaf tissue. **B**, Covalently bound proteins were eluted with 50 mM DTT and probed by immunoblot using α-SOS3 antibody. **C** and **D**, 2-BrP inhibits SOS3 import to the nucleus. **C**, Representative images of root meristematic epidermal cells of Arabidopsis *sos3-1* seedlings expressing *proSOS3:SOS3-GFP.* After 5 d growing under control conditions, plants were exposed for one additional day to mock or 50 µM of 2-BrP. Plants were treated with 0.1% Triton X-100 before imaging to allow counter-staining of nuclei with DAPI. Scale bar 5 µm. **D**, SOS3-GFP fluorescence signal (mean gray intensity) in the cytoplasm and nuclei of cells as shown in (C). Data are from 13 different plants, 10 cells each. Asterisks indicate that means were statistically different at *P* < 0.001; One-way Anova followed by Tukey's multiple comparisons test. **E**, Representative fluorescence images of root meristematic epidermal cells of *sos3-1* seedlings expressing *proSOS3:SOS3-GFP* under spinning disc confocal microscopy. Five-day-old seedlings were exposed for one additional day to the indicated treatments (mock, 100 mM NaCl, 50 µM 2-BrP, and 100 mM NaCl plus 50 µM 2-BrP). Arrows indicate the nuclei. Scale bar is 10 µm. **F**, Percentage of fluorescence intensity (mean gray intensity) after treatments normalized to the signal in the same cellular compartment under control conditions (mock) of samples shown in (E). Data are shown as box plots based on Tukey’s method: center lines show the medians; box limits indicate the 25th and 75th percentiles; whiskers length include values within 1.5 interquartile distance from the box. Triangles represent data points outside whiskers, and they were not excluded for statistical analyses. Data are from 9 different plants, 10 cells each; letters indicate significantly different means based on one-way analysis of variance followed by Tukey’s multiple comparisons test or Dunnett’s T3 test when variances were unequal (nucleus), *P* < 0.05. **G**, Nucleo-cytoplasmic fractionation. Nuclear and cytoplasmic proteins of *sos3-1* plants overexpressing WT SOS3, or mutants G2A (right panel) and C3A (left), were fractionated and probed with antibodies against SOS3, the cytoplasmic marker protein PEPC, and the nuclear marker Histone3 (H3). Loading of nuclear proteins was 10-fold higher than that of cytosolic proteins to compensate for the lower abundance of SOS3 in the nucleus.

Like GI, SOS3 is a nucleo-cytoplasmic protein (Batistic et al., 2010; Villalta et al., 2021). *N*-myristoylation of SOS3 at Gly-2 is essential for plasma membrane attachment and recruitment of SOS2 for salt tolerance, whereas *S*-acylation at residue Cys-3 promotes nuclear entry (Villalta et al., 2021). Therefore, we determined whether salt stress affected SOS3 *S*-acylation and tested the influence of *N*-myristoylation and *S*-acylation of SOS3 on salt-induced flowering delay. *S*-acylation at Cys-3 of WT SOS3 and mutant proteins G2A, C3A, and G2A/C3A expressed in *N. benthamiana* was tested by the acyl resin-assisted capture (acyl-RAC) method (Chai et al., 2019). Free cysteines in proteins were blocked with N-ethylmaleimide (NEM) prior to treatment or not with hydroxylamine (HyA), which breaks cysteine thioester bonds with fatty acids but not when cross-linked with NEM. Next, proteins were attached covalently to the resin matrix through the newly formed cysteine thiols by HyA treatment that had been protected by *S*-acylation against NEM binding. Proteins were considered to be *S*-acylated if retention was observed only upon HyA treatment. Results demonstrated that SOS3 was *S*-acylated at Cys-3 and that this modification took place independently of myristoylation of Gly-2 (Figure 4, A and B). The lower recovery of SOS3-G2A compared to SOS3 might be due to decreased accessibility of the nonmyristoylated protein to palmitoyl acyl-transferases (PATs), which are integral membrane proteins (Hemsley, 2020).

To analyze the *S*-acylation-dependent nuclear import of SOS3 in Arabidopsis, the *sos3-1* mutant was transformed with the construct *proSOS3:SOS3-GFP*, comprising a genomic copy of the *SOS3* gene designed to mimic the native *SOS3* gene expression. The functionality of this construct was validated by suppression of the salt sensitivity of the *sos3-1* mutant (Supplemental Figure S7). Treatment of these plants with the potent PAT inhibitor 2-bromo-palmitate (2-BrP) resulted in the complete exclusion of SOS3 from the nucleus (Figure 4, C and D). DAPI staining revealed that nuclear integrity was not noticeably affected by 2-BrP. Note that counter-staining with DAPI to visualize the nucleus required treatment with Triton-X100 to permeate the dye, which removed the SOS3-GFP signal at the plasma membrane. Next, we used spinning-disc confocal laser microscopy (SDCLM) to measure the relative amounts of SOS3-GFP at the plasma membrane, cytoplasm (comprising cytosol and endosomes) and nuclei (Figure 4, E and F). In SDCLM, multiplex laser excitation allows detection of the emission light at multiple points simultaneously for high-speed image acquisition and enhanced sensitivity towards low-abundance fluorescent proteins. Treatment with 2-BrP produced a statistically significant reduction in the nuclear pool of SOS3. Salinity (100 mM NaCl, 1 d) increased the abundance of SOS3 in all compartments, particularly in nuclei (Figure 4, E and F). The inhibitory effect of 2-BrP on the nuclear localization of SOS3 dominated over the stimulation by the saline treatment. Last, the nucleo-cytoplasmic partition of SOS3 was inspected in *sos3-1* plants transformed to express the SOS3 protein (*35S:SOS3*) with and without G2A and C3A mutations. Immunoblots with SOS3 antibodies of fractionated nuclear and cytoplasmic protein extracts demonstrated that protein SOS3-C3A was excluded from the nucleus whereas the nonmyristoylated SOS3-G2A mutant protein (which was still *S*-acylated; see Figure 4B) was imported into the nucleus (Figure 4G). Together, these data are evidence of the salinity-induced and *S*-acylation-dependent nuclear import of SOS3.

**Figure 5:**
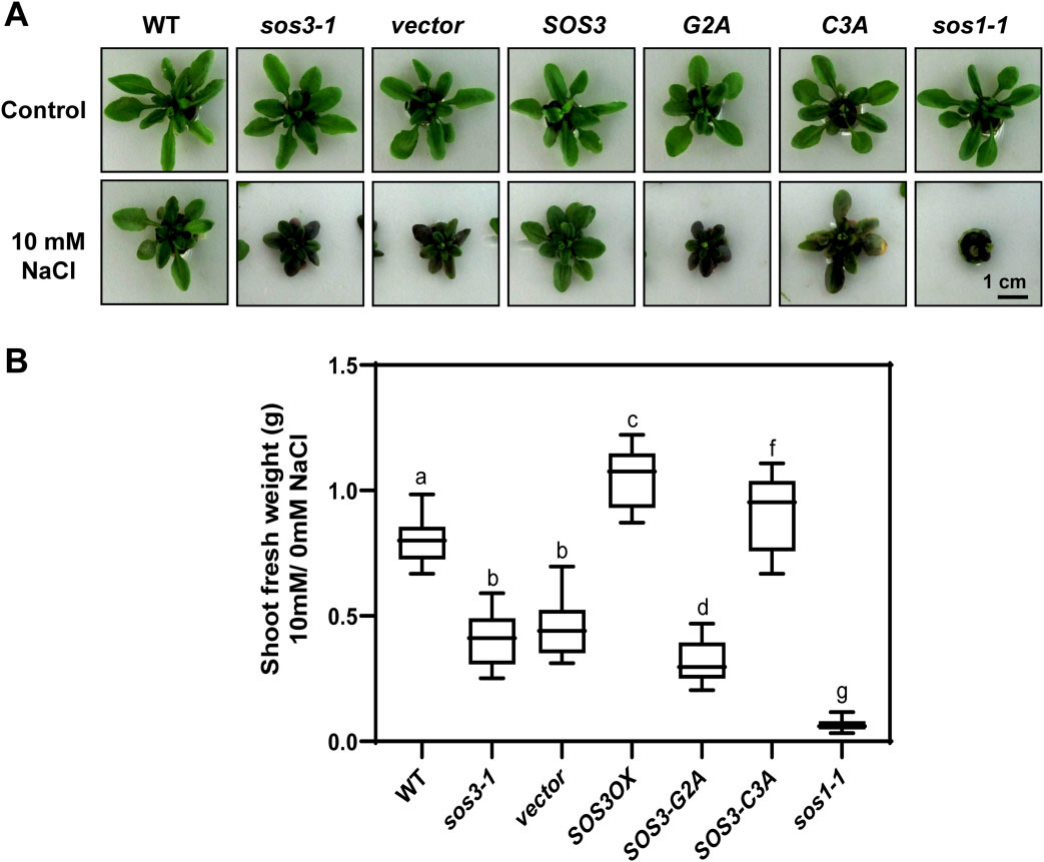
Nonpalmitoylated SOS3-C3A can partially rescue the salt sensitivity of *sos3-1.* A, Wild-type (Col-0 *gl1*; WT) and *sos3-1* plants, as well as *sos3-1* plants transformed with an empty vector (vector), over-expressing WT *SOS3* (*SOS3*), or producing nonmyristoylated SOS3 (*SOS3-G2A*) or nonpalmitoylated SOS3 (*SOS3-C3A*) were grown in hydroponics with LAK nutrient media with or without 10 mM NaCl. Selection of these transgenic lines is shown in Supplemental Figure S8. The *sos1-1* mutant plant was used as a salt-hypersensitive control. Photographs were taken after 2 weeks of salt treatment. B, Shoot fresh weight of plants shown in (A) was measured and normalized to that of nontreated plants. Data are shown as box plots: center lines show the medians; box limits indicate the 25th and 75th percentiles; whiskers extend to the minimum and maximum (*n* ≥ 9). Means followed by the same letter are not significantly different at *P* < 0.05 by the Fisher’s LSD test.

**Figure 6:**
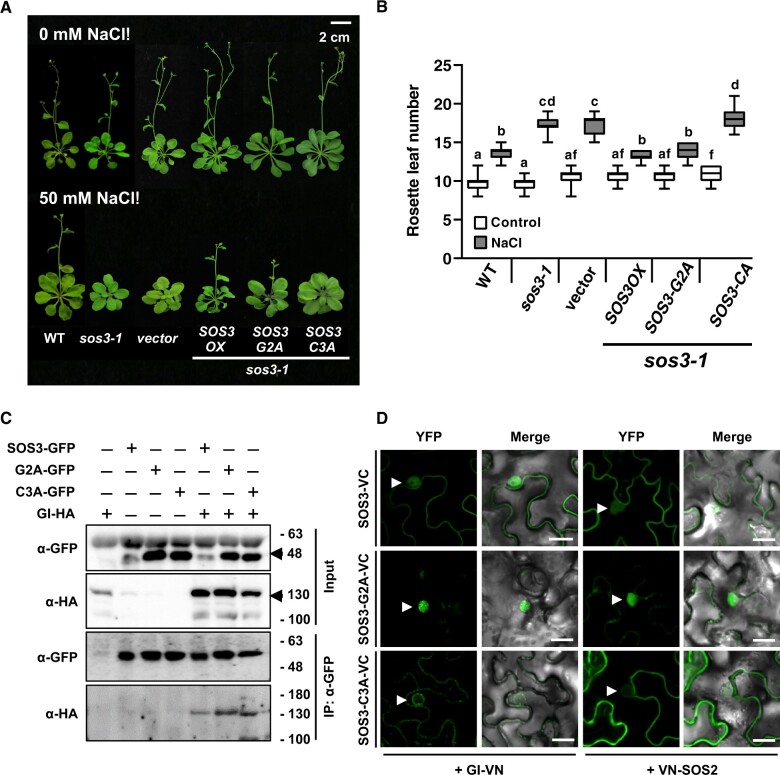
Nuclear localization of SOS3 is required for flowering under salt stress. A, Effect of salt on the flowering time of WT and mutant *sos3-1* plants, as well as *sos3-1* mutant plants overexpressing the WT *SOS3* (*SOS3-*OX) or producing nonacylated proteins SOS3-G2A and SOS3-C3A. Eight-day-old seedlings were transferred to MS media supplemented with 50 mM NaCl. Photographs were taken after bolting. B, Rosette leaf number was counted at bolting as an indicator of flowering time. Data are shown as box plots: center lines show the medians; box limits indicate the 25th and 75th percentiles; whiskers extend to the minimum and maximum (*n* = 15). Letters indicate significantly different means at *P* < 0.01 by Fisher’s LSD test. C, Co-immunoprecipitation of GI and SOS3 proteins. *SOS3-GFP*, *SOS3-G2A-GFP*, and *SOS3-C3A-GFP* were transiently co-expressed with *GI-HA* in *N. benthamiana* leaves. Total proteins were extracted and immunoprecipitation was done with GFP antibodies (α-GFP). Arrowheads indicate the target proteins. D, BiFC of mutant SOS3 proteins with GI (left) or SOS2 (right). Proteins were transiently expressed in *N. benthamiana* leaves and YFP signals were detected under a confocal microscope. Arrowheads indicate nuclei. Scale bar represents 20 µm.

We next tested the salt tolerance and flowering time of *sos3-1* transgenic lines expressing SOS3 protein variants with and without modifying fatty acids. As expected, the SOS3-G2A mutant failed to suppress the salt sensitivity of *sos3-1* plants in a hydroponics growth test (Figure 5). However, SOS3-C3A could partially rescue the hypersensitivity of *sos3-1*. The low abundance of the SOS3-C3A mutant protein compared to SOS3 and SOS2-G2A despite all plant lines having similar transgene transcript levels may explain the incomplete suppression (Supplemental Figure S8). Plants expressing SOS3, SOS3-G2A, and SOS3-C3A all flowered at a similar time under control conditions (Figure 6, A and B). However, upon salt treatment, plants expressing SOS3 and SOS3-G2A complemented the salt-induced flowering delay of *sos3-1* plants, but protein SOS3-C3A could not suppress this trait (Figure 6, A and B). These results indicate that nuclear import of *S*-acylated SOS3 is essential to its role in setting flowering time under salinity stress.

Next, we checked whether *N*-myristoylation and *S*-acylation of SOS3 were important for interaction with GI. Total proteins extracted from *N. benthamiana* leaves transiently expressing *GI-HA* together with *SOS3-GFP*, *SOS3-G2A-GFP*, or *SOS3-C3A-GFP* were used for co-IP. Both SOS3-G2A-GFP and SOS3-C3A-GFP were able to interact with GI protein similarly to SOS3-GFP (Figure 6C). When expressed in *N. benthamiana* leaves, SOS3-GFP and SOS3-G2A-GFP displayed a nucleo-cytoplasmic distribution, but the nuclear import of the SOS3-C3A-GFP mutant was suppressed (Supplemental Figure S9). Accordingly, GI formed BiFC complexes with SOS3 and SOS3-G2A inside the nuclei, but the nuclear interaction of GI with SOS3-C3A was severely reduced (Figure 6D and Supplemental Figure S10). *N*-myristoylation and *S*-acylation of SOS3 also affected the localization pattern of the SOS2-SOS3 BiFC complex (Figure 6D), indicating that the N-terminal modifications largely dictate the subcellular localization of the SOS3 protein complexes.

**Figure 7:**
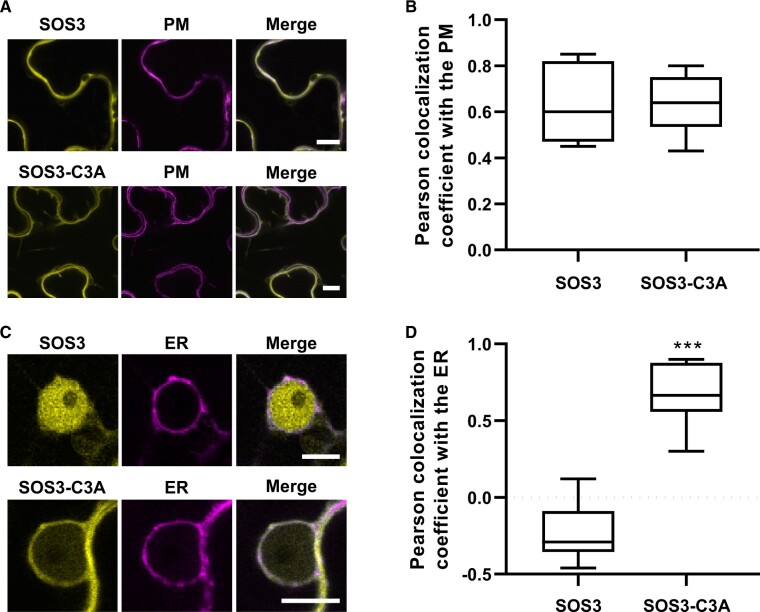
Subcellular distribution of nonpalmitoylated SOS3. A and B, Colocalization of SOS3-GFP and SOS3-C3A-GFP (in yellow) with the plasma membrane marker LTI6b:RFP (magenta) in *N. benthamiana* leaves. Scale bar represents 10 µm. C and D, Colocalization of SOS3-GFP and SOS3-C3A-GFP (yellow) with the endoplasmic reticulum marker RE:mCherry CD-393 (magenta) in *N. benthamiana* leaves. Scale bar represents 10 µm. B and D, Box plots showing the Pearson colocalization coefficient between SOS3 or SOS3-C3A with the markers; centerlines are the medians; box limits indicate the 25th and 75th percentiles; whiskers extend to the minimum and maximum; *n* ≥ 5 individual cells. Asterisks indicate significantly different means relative to the WT protein, based on Welch’s unpaired *t* test, *P* < 0.001.

The GI complex with SOS3-C3A localized in a perinuclear rim suggesting the retention of the complex in the perinuclear ER (Figure 6D and Supplemental Figure S10). Hence, we tested the fate and subcellular localization of the GFP-tagged SOS3-C3A protein by co-expression with plasma membrane and ER markers. The WT SOS3-GFP produced a bipartite localization comprising a cytosolic signal overlapping with the plasma membrane marker LTI6b:RFP as well as intense labeling of nucleoplasm (Figure 7, A and B). SOS3-C3A also had a bipartite localization with fluorescence arising from the plasma membrane, owing to preserved *N*-myristoylation, and from the ER, correlating with the ER-mCherry marker (Figure 7, C and D). These results suggest that a fraction SOS3-C3A is retained at the ER, which is coherent with the GI/SOS3-C3A complex remaining in a perinuclear rim encompassing the nuclear envelope and/or the ER, and strongly suggest that *S*-acylation of SOS3 dictates the localization of the SOS3-GI complex inside the nucleus.

### SOS3 interacts with GI and FKF1 to regulate *CO* expression under salt stress

**Figure 8:**
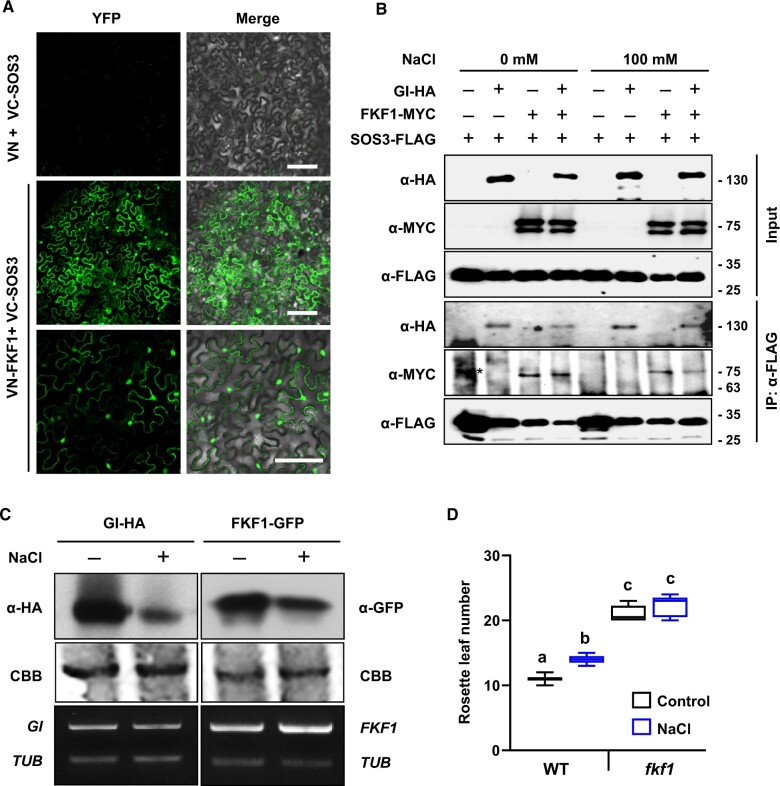
SOS3 interacts with FKF1. **A,** FKF1 and SOS3 were fused to the N- and C-termini of the Venus fluorescent protein and expressed into *N. benthamiana* leaves. Fluorescence was detected on the third day after infiltration. Scale bars are 100 µM. **B,** SOS3-FLAG was transiently co-expressed with GI-HA and/or FKF1-MYC in *N. benthamiana* leaves. Total proteins from leaves treated with 100 mM NaCl for 8 h were extracted and immunoprecipitated with FLAG antibodies (α-FLAG). (*), nonspecific band. The experiment was repeated 3 times with similar results. **C,** *GI* and *FKF1* overexpressing plants carrying *35S:GIHA* (*GI-HA*) and *35S:FKF1-GFP* (*FKF1-GFP*), respectively, were treated with 100 mM NaCl at ZT0. GI and FKF1 protein abundance was evaluated after 12 h NaCl treatment by immunoblotting with antibodies α-HA and α-GFP (upper panel). Coomassie brilliant blue (CBB)-stained blots are shown as a loading control (middle). Total RNA was extracted from *GI-HA* and *FKF1-GFP* plants and submitted to cDNA synthesis. *GI*, *FKF1*, and *TUBULIN* (*TUB*) transcript levels were evaluated by RT-qPCR (bottom). **D,** Eight-day-old WT and *fkf1* mutant plants were treated of not with 50 mM NaCl. The box plot shows the flowering time. Center lines indicate the medians; box limits indicate the 25th and 75th percentiles; whiskers extend to the minimum and maximum (*n* ≥ 5). Different letters indicate statistically significant differences between means at *P* < 0.01 by the Fisher’s LSD test.

FKF1 is a blue-light photoreceptor that mediates light-dependent protein degradation by the E3 ubiquitin ligase complex. FKF1 associates with GI to degrade CDF1, a *CO* transcriptional repressor that acts in the late afternoon of LDs (Suarez-Lopez et al., 2001; Imaizumi et al., 2005; Sawa et al., 2007). Therefore, we tested whether SOS3 interacted with FKF1. First, BiFC in *N. benthamiana* demonstrated that FKF1 and SOS3 protein associated physically in the cytosol and nuclei (Figure 8A). Next, tagged proteins GI-HA, FKF1-MYC, and SOS3-FLAG were co-expressed in *N. benthamiana* leaves and submitted to co-IP. SOS3 pulled down both GI and FKF1 under regular and saline conditions (Figure 8B). Similar to *gi* mutant plants, the *fkf1* mutant showed unconditional delayed flowering irrespective of the saline treatment. However, salinity had a lesser effect on FKF1 protein abundance compared to its effect on GI abundance (Figure 8, C and D). Together, these results demonstrate that SOS3 interacts individually with both GI and FKF1.

**Figure 9:**
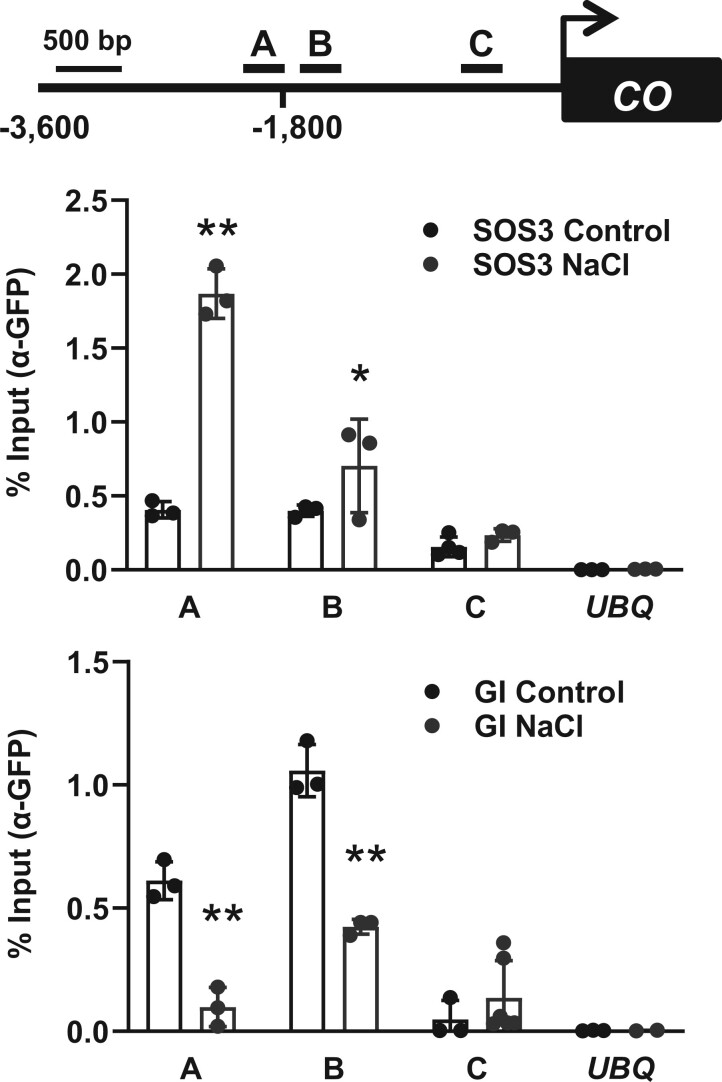
Salt-induced association of SOS3 with the *CO* promoter. Top, schematic drawing of the *CO* gene promoter and locations of amplicons (A, B, and C) used for ChIP analysis. Chromatin isolated from 2-week-old *SOS3-GFPox* and *GI-GFPox* plants treated with 100 mM NaCl (NaCl) for 10 h or not (Control) was immunoprecipitated with α-GFP antibodies. Immunoprecipitated and input DNA were used as templates for qPCR using primers specifically targeting the amplicons A, B, and C. *UBQ10* was used as a control. Data show fragment enrichment as a percent of input DNA. Error bars represent SE of technical replicates; individual values are shown. Asterisks indicate statistically significant differences between control and treated samples at *P* < 0.01 (**) or *P* < 0.05 (*) by Fisher’s LSD test. The experiment was repeated twice with similar results.

The FKF1-GI complex associates with the *CO* promoter to induce flowering (Sawa et al., 2007), and our evidence that SOS3 interacted physically with these proteins suggested that SOS3 could be present at the transcriptional complex regulating *CO* transcription. A chromatin-immunoprecipitation (ChIP) analysis of *sos3-1* transgenic plants expressing *SOS3-GFP* showed a salt-dependent enrichment of SOS3-GFP in the same *CO* promoter region where GI and FKF1 mostly associate (amplicon A, Supplemental Figure S11; Sawa et al., 2007). SOS3-GFP was largely absent from the *CO* promoter under nonsaline control conditions. Fragment C of the *CO* promoter with no GI binding, the *UBQ10* promoter, and nontransformed plants were used as negative controls. To compare the salt-induced changes in *CO* promoter occupancy by GI and SOS3, WT plants expressing GFP-tagged GI and SOS3 were treated or not with NaCl before the ChIP assay. Again, SOS3-GFP association with amplicon A of the *CO* promoter increased 4- to 5-fold upon NaCl treatment (Figure 9 and Supplemental Figure S11). By contrast, the saline treatment reduced GI abundance in the *CO* promoter, likely reflecting the instability of cellular GI under these stress conditions (Figure 9). Note that the net amount of immunoprecipitated protein-chromatin complexes expressed as a percent of input cannot be directly compared for SOS3 and GI, owing to the different efficacy of target protein immunoprecipitation; only differences between treatments should be considered for each protein. Together, these results indicate that *S*-acylation enables nuclear translocation of SOS3 to associate with the GI-FKF1 complex to control *CO* expression under salt stress, thereby affecting flowering time.

## Discussion

### Salt stress and flowering time

Plants adjust their transition from vegetative growth to reproduction by constantly monitoring and integrating environmental cues. Water or nutrient deprivation often leads to earlier flowering presumably because the lack of essential resources inevitably halts growth, whereas a transitory stress, such as salinity, is more likely to postpone flowering so that reproduction can resume at a later time (Kazan and Lyons, 2016; Maggio et al., 2018). Stress-induced early flowering is an emergency response to proceed to the next generation when vegetative plants cannot cope with adverse environmental conditions (Takeno, 2016). For instance, the drought-escape response entails adaptive shortening of the vegetative growth phase and anticipated seed production before severe dehydration becomes lethal (Riboni et al., 2013). By contrast, salinity delays flowering in several species, including Arabidopsis (Kim et al., 2007b, 2013a; Li et al., 2007; Ryu et al., 2014). Plausibly, this reproductive strategy reflects that nonlethal saline levels reduce but do not impede vegetative growth since plants have developed adaptive strategies to overcome both the osmotic and ionic stresses imposed by salinity (van Zelm et al., 2020). In this regard, the ubiquitous SOS pathway enables plants to deal with excess Na^+^ through the coordination of ion fluxes back to the soil solution and into the xylem to protect roots from damage (Ji et al., 2013; El Mahi et al., 2019). We suggest that the ecophysiological meaning of salt-induced flowering delay is to allow plants to acclimate by simultaneously reducing their growth rate and altering the developmental program to extend the vegetative growth phase long enough to gather sufficient metabolic resources to ensure robust flowering and seed filling. From an evolutionary perspective, it is beneficial that the control of flowering time and the physiological response to salinity stress are molecularly linked (Kazan and Lyons, 2016). We show that in Arabidopsis the reciprocating mechanism coordinating these signaling networks occurs through the physical interaction and mutual regulation of GI, SOS2, and SOS3, to simultaneously mount salt tolerance and postpone reproduction. That gibberellin GA4 counteracted salinity-induced late flowering (Li et al., 2007) supports the notion that delayed flowering is a pro-active, genetically ingrained stress response partly dependent on DELLA proteins (Achard et al., 2006).

### Flowering under saline stress requires GI stabilization by SOS3 in the nucleus

Salinity-induced delay in the flowering time of Arabidopsis occurs *via* reduced transcription of the floral integrators *CO* and *FT* in a dosage dependent manner (Kim et al., 2007b; Li et al., 2007). In control conditions, the *co-2* mutant displayed late flowering. Salinity delayed flowering in a dose response manner both in WT and *co-2* mutant plants, with a greater progression in the WT so that both genotypes reached a similar retardation at 100 mM NaCl (Li et al., 2007). The GI protein, a major regulator of photoperiodic-induced flowering through the CO-FT module, also plays a substantial role as a negative regulator of salt stress adaptation by sequestering the SOS2 kinase (Kim et al., 2013a). Despite the multiplicity of SOS2 functions in various processes pertinent to adaptation to salinity (Ji et al., 2013), SOS2 does not seem to play a significant role in setting the flowering time of Arabidopsis on its own. However, our study reveals that SOS3, a critical regulator of SOS2, does modulate the initiation of flowering under salt stress by binding to and stabilizing GI in the nucleus (Figure 2). Nuclear localization of the GI-SOS3 complex was abolished in plants expressing the non-*S*-acylatable SOS3-C3A protein that remained outside the nucleus (Figures 4 and 7; Supplemental Figures S9 and S10). Since only the nucleus-localized GI is competent to promote flowering (Kim et al., 2013b), these results indicate that SOS3 promotes the stabilization of nuclear GI during salt stress and prevents further delay in flowering.

Among the 10 CBL proteins of Arabidopsis, dual fatty acid modifications consisting of *N*-myristoylation and *S*-acylation contribute to the cellular sorting of CBL1, SOS3/CBL4, CBL5, and CBL9 (Batistic et al., 2008; Batistic et al., 2010). *N*-myristoylation of SOS3 at Gly-2 allows the SOS2-SOS3 complex to associate with the plasma membrane and phosphorylate SOS1 to promote Na^+^ efflux (Quintero et al., 2002; Villalta et al., 2021). *S*-acylation of the cysteine residue adjacent to the myristoylated glycine is thought to enhance the membrane attachment of CBL proteins and to regulate subcellular trafficking from the ER to the plasma membrane (Batistic et al., 2008; Held et al., 2011; Saito et al., 2018). The CBL4-CIPK6 complex modulates the K^+^ channel function of AKT2 by promoting its sorting from the ER to the plasma membrane (Held et al., 2011). Mutants in each of the three components of this functional module exhibit delayed flowering only in SD conditions but not in LDs, but the floral regulators altered in these mutants were not investigated and the precise molecular connection between K^+^ status and flowering time remained unknown (Held et al., 2011). Here, we show that *S*-acylation of SOS3 is a requisite for nuclear import to regulate flowering time under LD and saline conditions. Mutation of the *S*-acylation site (SOS3-C3A) or biochemical inhibition with 2-BrP impeded translocation to the nucleus and relocalized the complex SOS3-GI to the perinuclear ER. However, SOS3-C3A was able to partially suppress the salt-sensitivity of mutant *sos3-1*, whereas the nonmyristoylatable mutant SOS3-G2A could not restore salt tolerance but supported normal flowering. This indicates that *S*-acylation of SOS3 is specifically and critically important for resetting flowering time under salt stress.

### *S*-acylation-dependent nuclear entry of SOS3

Eukaryotic PATs of the DHHC (Asp-His-His-Cys) family catalyze protein *S*-acylation (Batistic, 2012; Hemsley, 2020). In turn, thioesterases break down the ester bond of *S*-acylation and release the fatty acid. The unique reversibility of protein *S*-acylation allows proteins to rapidly change their location between intracellular compartments (Aicart-Ramos et al., 2011; Chamberlain and Shipston, 2015; Hemsley, 2020). For instance, the stress-responsive transcription factor NFAT5a is *N*-myristoylated, *S*-acylated and sorted to the plasma membrane of animal cells. Upon osmotic stress, NFAT5a moves into the nucleus by a mechanism involving de-S-acylation (Eisenhaber et al., 2011). In plants, the *N. benthamiana* protein HIPP26 acts as a plasma membrane-to-nucleus signal during abiotic stress and viral infection. Lipidation (*S*-acylation and prenylation) recruits HIPP26 to the plasma membrane and plasmodesmata, but interference of HIPP26 *S*-acylation by the interacting viral protein TGB1 results in nuclear accumulation of the nonlipidated HIPP26 protein (Cowan et al., 2018). In these examples, conditional *S*-acylation serves as a lipid anchor at membranes to immobilize and restrain proteins from entering the nucleus until they are de-*S*-acylated, which is in contrast to the results we report here for SOS3 nuclear import. To date, there is no molecular mechanism known to promote the nuclear import of a lipid-modified protein in an *S*-acylation-dependent manner (Aicart-Ramos et al., 2011; Chamberlain and Shipston, 2015; Hemsley, 2020). SOS3 may enter the nucleus assisted by a shuttle or gateway protein, whose interaction is presumably dependent on the *S*-acylation status of SOS3. The finding that non-S-acylated SOS3 was still able to interact with GI and that the complex was detected at the nuclear rim discards the possibility that GI shuttles SOS3 to the nucleus as a complex. While SOS3 *S*-acylation is a strict requirement for nuclear recruitment, it remains unknown whether SOS3 undergoes de-*S*-acylation when entering the nucleus or is processed therein (Villalta et al., 2021). Quantitative data in Figure 4 indicate that salinity enhances *S*-acylation and the transfer of SOS3 to the nuclear pool relative to the whole cell SOS3 content. Understanding the environmental and biochemical inputs that elicit *S*-acylation (and de-*S*-acylation) of SOS3, and identifying the PATs localized in the nuclear envelope or in the perinuclear ER membrane involved in this process is a promising line of research.

### SOS3 ensures GI-mediated flowering under salt stress

Here, we show that nuclear GI is more recalcitrant to degradation than cytosolic GI, presumably by stabilization of the GI-SOS3 complex inside the nucleus. However, GI shuttles dynamically between the cytosol and nucleus (Kim et al., 2013b), and we could reasonably assume that depletion of the cytosolic GI pool under saline stress may detract from the nuclear pool, thereby reducing over time the amount of GI available for *CO* transcription and delaying flowering. The dose- and time-dependent decay of *CO* and *FT* transcript abundance supports this assumption (Supplemental Figure S2). GI is a large plant-specific protein considered to be a scaffold or co-chaperone (Cha et al., 2017); its activity is modulated by many physiological effectors. The selective binding of different partnering proteins determines GI-dependent outputs in several physiological processes. GI is component of the evening loop in the circadian clock (Park et al., 1999). Moreover, and in association with SOS1, GI sustains a proper clock period under salinity conditions (Cha et al., 2022). In the photoperiodic flowering pathway, GI associates with FKF1 and ZTL, which control both the subcellular partition of GI and binding to the *CO* promoter. GI can also associate with TCP4 to bind to the *CO* promoter (Kubota et al., 2017). Last, the *CO* promoter itself is busily occupied by BHLHs, CDFs and a plethora of chromatin remodeling proteins that control *CO* transcription during the evening of a LD (He et al., 2020). Therefore, recruiting of GI to the *CO* promoter, which here we show is influenced by salt, could be a separate factor from its concentration in the nucleus, as the role of GI as a transcriptional co-factor is regulated by a rather complex mechanism involving many other proteins. We envision that several other factors controlling GI occupancy of the *CO* promoter under saline stress will surely be at play in addition to SOS3.

Since GI and FKF1 are not DNA-binding proteins per se, the abundance or regulated activity of other transcriptional players under different environmental conditions might affect the effectiveness of the GI-FKF1 complex in promoting *CO* expression. CO acts as a network hub that integrates external and internal signals into the photoperiodic flowering pathway (Park et al., 2016; Shim et al., 2017). Consequently, the transcription and post-transcription of CO is complex, with the concourse of multiple positive and negative effectors balancing reciprocally. We show here that a novel function of SOS3 is to adjust flowering time to a saline environment. Presumably, SOS3 provides information about environmental stressors as much as GI inputs photoperiodic information to fine-tune flowering time with a given environment. The logic of why a stress protein like SOS3 should have a positive effect in setting flowering time when this developmental transition is being delayed by salinity stress remains obscure at this time. However, our data clearly demonstrate that nucleus-localized SOS3 positively regulates flowering time specifically under salinity stress.

Ultimately, the developmental transition to flowering requires derepression of *CO* and *FT*. Expression of *CO* under LD requires the degradation of repressors collectively known as CDFs by a protein complex formed by GI and FKF1 (Imaizumi et al., 2005; Sawa et al., 2007; Fornara et al., 2009). Even though SOS3 was able to interact with GI and FKF1 both in normal and saline conditions (Figure 8), the association of SOS3 to the GI and FKF1 binding sites in the *CO* promoter increased upon salt treatment, thereby leading to enhanced *CO* expression upon the onset of salt stress (Figure 1C). This result and the spinning disc confocal microscopy data (Figure 4, E and F) support the notion that salt stress enhances nuclear import of SOS3 through *S*-acylation to promote the expression of *CO*. We have not yet investigated whether CDF repressors are displaced or degraded upon binding of the GI-FKF1-SOS3 complex.

**Figure 10:**
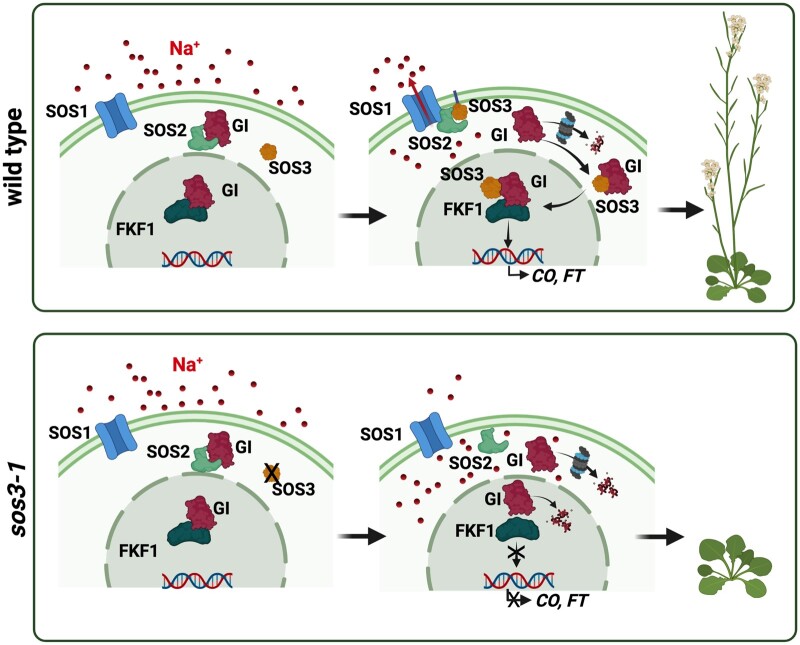
Working model. Upon salt stress, SOS3 senses and binds elevated cytosolic Ca^2+^. Calcium-bound SOS3 activates and recruits SOS2 to the plasma membrane through the myristoylation of SOS3 to phosphorylate and activate SOS1, a Na^+^ transporter mediating ion exclusion and salt tolerance. Free cytosolic GI is degraded. *S*-acylated SOS3 enters the nucleus and, together with GI and FKF1, supports *CO* expression to regulate flowering time under salt stress. Created with BioRender.com

Here, we have analyzed the diurnal pattern of *CO* and *FT* transcription and protein abundance upon the onset of saline treatment, coinciding with the beginning of the enhanced nuclear import of SOS3 (Figures 1 and 4). *CO* transcription and CO protein abundance are known to be under complex multilayered regulation (Shim et al., 2017), and the expression pattern is susceptible to change along the process of salt-adaptation. Indeed, quantitation of *CO* and *FT* transcripts at Days 1 and 5 showed that the salt- and genotype-dependent reduction in *FT* expression was more intense after 5 d in salt compared to after 1 d, and that *CO* transcript abundance showed a similar trend after the 5-d treatment (Supplemental Figure S2). The negative impact of the *sos3-1* mutation was observed regardless of the duration of the salt treatment. The early increase in the abundance of *CO* transcripts in the WT upon salt treatment and the opposing decrease in the *sos3-1* mutant is coherent with the model depicted in Figure 10. In the WT plant, the nuclear pool of GI is not degraded but protected by the nucleus-imported SOS3, which together with FKF1 up-regulates *CO* transcription. However, in the *sos3-1* mutant, nuclear GI is also degraded and *CO* transcription is compromised. What ultimately determines floral commitment is the abundance of the florigen FT, and our *FT* expression and FT protein abundance data are in agreement with delayed flowering in the WT and the acute delay in the *sos3-1* mutant (Figure 1 and Supplemental Figure S2). Although CO is a major transcriptional activator of *FT*, GI also promotes *FT* expression in a CO-independent manner (Jung et al., 2007; Sawa and Kay, 2011). Because GI protein abundance decreased the most in the *sos3-1* mutant under salt stress, a condition that reduced *CO* transcription and completely abated *FT* transcripts, it appears that *FT* transcription under saline stress is largely determined by both GI and CO abundance. It remains to be investigated whether the GI-FKF1-SOS3 complex also regulates *FT* transcription directly as with *CO*.

The model depicted in Figure 10 summarizes the stress-related functions of SOS3. A salt-induced Ca^2+^ signal activates SOS3 and fosters the interaction with GI (Figure 3). A fraction of the SOS3 protein is *S*-acylated and the SOS3-GI complex is partitioned into the nucleus to regulate flowering time (Figure 4), whereas the *N*-myristoylated but non-*S*-acylated SOS3 recruits SOS2 to the plasma membrane to activate SOS1 (Figures 4 and 5; Villalta et al., 2021). This model adds new layers of regulation and molecular connections to the mechanism that links salt stress adaptation and the photoperiodic flowering pathway, exemplifying the plasticity inherent to plant development. Our results also uncover a potentially novel mechanism for palmitoylation-dependent ingress of proteins into the nucleus.

## Materials and methods

### Plant materials

Arabidopsis (*A. thaliana*) lines *sos1-1 GI-HA*, *sos2-2 GI-HA*, *sos3-1 GI-HA*, and *cbl10 GI-HA* were generated by crossing *GI-HA* transgenic plants (David et al., 2006) with the mutants *sos1-1, sos2-2, sos3-1*, and *cbl10* (Zhu et al., 1998; Quan et al., 2007). Plants were confirmed for salt sensitivity and verified for *GI* expression. The *sos* mutant lines had levels of transgene expression and GI-HA protein abundance similar to the original *GI-HA* line (Supplemental Figure S12). Transgenic lines *proGI:GI-GFP* (called *GI-GFP*), *proGI:GI-GFP-NLS* (*GI-NLS*), and *proGI:GI-GFP-NES* (*GI-NES*) in the *gi-2* mutant background have been described elsewhere (Kim et al., 2013b). Transgenic lines expressing *SOS3*, *SOS3-G2A*, *SOS3-C3A*, and *SOS3-GFP* from the *35S* promoter and the genomic construct *proSOS3:SOS3-GFP* were generated in the *sos3-1* background (further details are in the Supplemental Methods). Functionality of these SOS3 constructs was tested for transgene expression and/or complementation of the salt-sensitive phenotype of the *sos3-1* mutant (Supplemental Figures S7 and S8).

### Flowering time under salinity stress

All seeds were sterilized with 70% ethanol and 2% bleach (sodium hypochlorite solution) and stratified at 4°C for 2–3 d. Plants were grown under LD conditions (16 h light/8 h dark, 80–100 μM m^−2^s^−1^) at 23°C. For flowering phenotype seeds were first grown on full MS media (Duchefa) containing 1% sucrose (supplemented with vitamins, 2.5% phytagel). Then 8-d-old seedlings were transferred to MS media (basal salt MS without vitamins) with or without NaCl in plant culture dishes (14 cm in height; 10 cm in diameter). Six plants were planted per plant dish with sufficient air exchange. Salt-treatments were adjusted depending on the genotype and the relative sensitivity or tolerance to salinity of plants used in each experiment, to ensure that plants survived the treatment and flowered. The concentrations of NaCl used are indicated in the figure legends. Total rosette leaf numbers were counted after bolting to measure flowering time.

### Hydroponics for salt tolerance phenotypes

Seeds were sterilized, stratified for 2 d at 4°C, and then placed onto rockwool in hydroponic culture with aerated nutrient solution (LAK medium, 1 mM KH_2_PO_4_, 2 mM Ca(NO_3_)_2_, 1 mM MgSO_4_, 30 μM H_3_BO_3_, 10 μM MnSO_4_, 1 μM ZnSO_4_, 1 μM CuSO_4_, 0.03 μM (NH_4_)_6_Mo_7_O_24_, and 100 μM Fe^2+^ as Sequestrene 138-Fe, pH ∼5.3; Barragán et al., 2012) with or without supplemental NaCl. Photographs were taken 2–3 weeks after salt treatment.

### Biochemical methods

Standard procedures were followed for immunoblotting and immunoprecipitation of proteins. Total proteins were obtained in extraction buffer (100 mM Tris-HCl, pH 7.5, 150 mM NaCl, 0.5% NP-40, 1 mM EDTA, 3 mM DTT, and protease inhibitors 1 mM PMSF, 5 µg/mL leupeptin, 1 µg/mL aprotinin, 1 µg/mL pepstatin, 5 µg/mL antipain, 5 µg/mL chymostatin, 2 mM sodium orthovanadate [Na_3_VO_4_], 2 mM sodium fluoride [NaF], and 50 µM MG132; Kim et al., 2013a). For Arabidopsis protein extraction, 1% NP-40 was added to the extraction solution.

For GI protein blots in Arabidopsis, total proteins were extracted from 2-week-old plants of genotypes *GI-GFP*, *GI-NLS* and *GI-NES* treated with or without 100 mM NaCl for 6 and 12 h. For CO and FT protein blots, Col-0 and *sos3-1* plants were grown on 1/2 MS media (1% sucrose, pH 5.7, 7% agar) at 23°C under LD. Eight-days-old plants were treated with 100 mM NaCl in the same media at ZT1 (1 h after light-on), and harvested at ZT4 and ZT16 for two consecutive days. Total protein was extracted using the TRIzol (Invitrogen) protocol. Twenty-five microgram protein samples were resolved in 12% (v/v) acrylamide SDS-PAGE and transferred to PVDF membranes. Blots were blocked with methanol and incubated overnight at 4°C with 1:1,000 rabbit α-CO and α-FT antibodies as described (Serrano-Bueno et al., 2020). The specificity of α-CO and α-FT antibodies was assessed with protein extracts from plants of genotype *co-10* and *ft-10* (Supplemental Figure S13). Blots were visualized in a Chemidoc Imaging System using Clarity Western ECL substrate (Bio-Rad). Images were analyzed with the image Lab software (Bio-Rad). For *N. benthamiana*, proteins GI-HA, SOS2-GFP, SOS3-MYC and MYC-SOS3-1 were transiently expressed in leaves alone or in given combinations by *Agrobacterium* infiltration. Leaves were treated or untreated with 100 mM NaCl or 3 mM CaCl_2_. EGTA (2 mM) was used as a calcium chelator to inhibit calcium signaling. Immunoblotting followed published procedures (Kim et al., 2013a).

For immunoprecipitation, rat α-HA (1:250, Roche, #11867423001) or rabbit α-GFP polyclonal (1:250, Invitrogen, #A11120) antibodies were preincubated with protein A agarose (Invitrogen) at 4°C for 30 min. Protein extracts were added and further incubated for 1 h at 4°C. Complexes were separated by SDS–PAGE. Each immunoblot was incubated with the appropriate primary antibody (α-HA (1:2,000), α-GFP (1:5,000, Abcam, #ab6556,), α-HSP90 (1:50,000; Kim et al., 2013a), or α-MYC (1:1,000, Cell Signaling Technology, #2276) for 1 to 2 h at room temperature or overnight at 4°C. The antigen protein was detected by chemiluminescence using ECL-detecting reagents as above.

To determine the subcellular localization of SOS3, SOS3-G2A and SOS3-C3A proteins, aerial parts of 4-week-old *sos3-1* plants expressing these proteins were collected for fractionation of nuclear and cytosolic proteins. Nuclei were purified using a Plant Nuclei Isolation/extraction Kit (Sigma). Cytosolic proteins were precipitated from supernatants of the first nuclei pelleting step with 10% TCA and resuspended in a denaturing buffer consisting of 50 mM Tris-HCl (pH 7.8), 4 M urea, 2% SDS and 2.5% glycerol. Commercially available antibodies against α-Histone3 (Abcam, #ab1791) and α-Phospho Enol Pyruvate Carboxylase (PEPC; Agrisera, #AS09 458) were used as nuclear and cytosolic markers. For the acyl resin-assisted capture (acyl-RAC) of SOS3, the WT protein and mutants G2A, C3A and G2A/C3A were expressed in *N. benthamiana* leaves. Protein samples from leaf tissue were divided into two parts and HyA was added at 0.5 M final concentrations to one of these parts to break *S*-acyl-thioester bonds, whereas the other served as an untreated control. Each sample was incubated with thiopropyl-sepharose 6B resin (Sigma) to link proteins with free thiols. Proteins eluted with a DTT-containing buffer were analyzed by immunoblotting with α-SOS3 antibody. Before sample processing, aliquots were withdrawn and kept as “input”. Further details are given in the Supplemental Methods.

### Microscopy

Plasmid constructs for BiFC and regular confocal microscopy are described in detail in the Supplemental Methods. For the 2-bromo-palmitate treatment and spinning-disc confocal microscopy, plants were sown in ½ MS plates with 1% sucrose in an LD chamber at 21°C. Five-day-old seedlings were incubated in the same liquid media with or without 50 µM 2-bromopalmitate (2-BrP; Sigma-Aldrich), and with or without 100 mM NaCl, for 24 h. A mock treatment with the same volume of ethanol (2-BrP solvent) was added to control samples. For spinning-disc confocal microscopy, images were generated using a confocal microscope equipped with a 113 CSU-W1 spinning disc head (Yokogawa) fitted to a Nikon Eclipse Ti-E-inverted microscope with a CFI PlanApo VC 60× N.A. 1.40 oil immersion objective and an EM-CCD ImageEM 1K (c9100-14) camera. GFP was imaged using a 488-nm solid-state diode laser and a 525/50-nm emission filter. The fluorescence intensity (mean gray value, arbitrary units) of *proSOS3:SOS3GFP* plants was measured in different cellular compartments. Image processing was done with Fiji software, version 1.57. A Gaussian blur filter (radius 1 px) was applied for the representative pictures. Details about regular confocal microscopy in the 2-BrP experiment are given in the Supplemental Methods.

### RNA isolation and RT-qPCR

Gene expression was analyzed by reverse transcription quantitative PCR (RT-qPCR) as detailed in the Supplemental Methods. Each data point represents the average of three independent amplifications of the same RNA sample run in the same reaction plate. Each biological replicate had three technical replicas. Primers used for RT-qPCR are in Supplemental Table S1.

### ChIP assay

Two-week-old Arabidopsis seedlings (*GI-GFPox* and *SOS3-GFPox*) treated with 100 mM NaCl for 10 h were used for the ChiP assay. Procedures of fixation and isolation of chromatin were performed as described (Sawa et al., 2007; Saleh et al., 2008). Two-week old Arabidopsis seedlings (*GI-GFPox* and *SOS3-GFPox*) treated with 100 mM NaCl for 10 h, and then fixed with 1% formaldehyde under vacuum for 15 min. Subsequently 0.125 M glycine was added to stop cross-linking. Nuclei were isolated in nuclei isolation buffer (0.25 M sucrose, 15 mM PIPES pH 6.8, 5 mM MgCl_2_, 60 mM KCl, 15 mM NaCl, 1 mM CaCl_2_, 0.9% Triton X-100, 1 mM PMSF, 2 μg/mL pepstatin A, and 2 μg/mL aprotinin), lysed in ice-cold nuclei lysis buffer (50 mM HEPES pH 7.5, 150 mM NaCl, 1mM EDTA, 1% SDS, 0.1% sodium deoxycholate, and 1% Triton X-100, 1 μg/mL pepstatin A, and 1 μg/mL aprotinin), and sonicated to shear DNA to approximately 500 bp. The chromatin samples were diluted 10-fold in lysis buffer and incubated with salmon sperm DNA/protein A agarose bead (Millipore) for 1 h. Appropriate antibody (α-GFP, Abcam, #ab290) was added to the supernatants and incubated overnight. The beads were washed with ice cold low salt wash buffer (150 mM NaCl, 20 mM Tris-HCl, pH8, 0.2% SDS, 0.5% Triton X-100, and 2 mM EDTA), high salt wash buffer (500 mM NaCl, 20 mM Tris-HCl, pH8, 0.2% SDS, 0.5% Triton X-100, and 2 mM EDTA), LiCl wash buffer (0.25 M LiCl, 1% sodium deoxycholate, 10 mM Tri-HCl pH8, 1% NP-40, and 1 mM EDTA), and TE buffer (1 mM EDTA and 10 mM Tris-HCl pH8) sequentially. Immunocomplexes were eluted with elution buffer (0.5% SDS and 0.1 M NaHCO_3_) at room temperature. Reverse-crosslinking was done by incubating with NaCl overnight at 65°C and subsequent incubation with proteinase K. DNA was purified by phenol-chloroform extraction and rescued by ethanol precipitation with 20 μg glycogen (Fermentas). DNA was resuspended in 50 μL TE buffer and 2 μL aliquots were used for qPCR. The primers used in this experiment are in Supplemental Table S1.

### Statistical analyses

The statistical analyses used to obtain the significance levels are indicated in the legends to each figure. The different statistical analyses were performed using GraphPad Prism version 8, GraphPad Software.

## Accession numbers

GIGANTEA, At1g22770; FKF1, At1g68050; CONSTANS, At5g15840; FT, At1g65480; SOS1/NHX7, At2g01980; SOS2/CIPK24, At5g35410; SOS3/CBL4, At5g24270; CBL10/SCaBP8, At4g33000.

## Supplemental data

The following materials are available in the online version of this article.

**Supplemental Figure S1.** Expression under salt stress of genes regulating flowering.

**Supplemental Figure S2.** Expression of *CO* and *FT* at 1- and 5-d under salt stress.

**Supplemental Figure S3.** Flowering phenotype of *sos3-1 gi-1* double mutant plants.

**Supplemental Figure S4.** Salt induced degradation of GI protein occurs in cytosol.

**Supplemental Figure S5.** CBL10 is not involved in the salt-induced late flowering.

**Supplemental Figure S6.** BiFC of SOS3 and GI in *N. benthamiana* leaves.

**Supplemental Figure S7.** Functional validation of the *proSOS3:SOS3:GFP* construct.

**Supplemental Figure S8.** Nonpalmitoylated SOS3-C3A can partially rescue the salt sensitivity of *sos3-1.*

**Supplemental Figure S9.** Subcellular distribution of nonpalmitoylated SOS3.

**Supplemental Figure S10.** Nonpalmitoylated SOS3 fails to interact with GI in the nucleus.

**Supplemental Figure S11.** SOS3 associates with the *CO* promoter.

**Supplemental Figure S12.** Equal *GI* gene expression in *sos1,2,3* mutants.

**Supplemental Figure S13.** Specificity of CO and FT antibodies.

**Supplemental Table S1.** List of primers used in this study and their purpose.

**Supplemental Table S2.** List of constructs used in this study.

**Supplemental Data Set S1.** Statistical analyses

## Supplementary Material

koac289_Supplementary_Data

## Notes

H.J.P., F.G.-A., M.L., R.A.B., S.Y.L., F.V., C.S.-R., J.M.P., W.-Y.K., F.J.Q., and D.-J.Y. designed the research. H.J.P., F.G.-A., M.L., R.A., I.V., J.-Y.C., R.C., C.J.L., E.G., F.V., and F.J.Q. performed the experiments. H.J.P., F.G.-A., F.V., C.S.-R., J.M.P., W.-Y.K., F.J.Q., and D.-J.Y. analyzed the data. H.J.P., F.G.-A., J.M.P., F.J.Q., and D.-J.Y. wrote the article.

The authors responsible for distribution of materials integral to the findings presented in this article in accordance with the policy described in the Instructions for Authors (https://academic.oup.com/plcell) are: Francisco J. Quintero (fjquintero@ibvf.csic.es) and Dae-Jin Yun (djyun@konkuk.ac.kr).
