# Supplementary material for: S-acylated and nucleus-localized SALT OVERLY SENSITIVE3/CALCINEURIN B-LIKE4 stabilizes GIGANTEA to regulate Arabidopsis flowering time under salt stress
Source: Plant Cell. 2022 Sep 22;35(1):298–317. doi: 10.1093/plcell/koac289 (PMC9806564; doi:10.1093/plcell/koac289)
Supplement: koac289_Supplementary_Data [file koac289_supplementary_data.zip › SupplementaryFile/TPC2022RA00153R2_Supplemental_Figures_and_Tables.pdf]

SUPPLEMENTAL METHODS

Plasmid constructs

For BiFC, full-length ORF sequences for *SOS2* and *SOS3* were amplified with indicated primers (Supplemental Table S1) to generate entry vectors with or without stop codons in the *pDONR*^{TM}*/Zeo* vector (Invitrogen, Carlsbad, CA, USA). cDNAs of *GI*, *SOS3* and *SOS3-1* were cloned under the *35S* promoter into vectors pDEST-^{GW}VYNE for *GI-VN,* and in pDEST-^{GW}VYCE for *SOS3-VC* or *SOS3-1-VC*, respectively, using Gateway cloning system. The SOS3-1 protein bears a three amino acid deletion in the third EF-hand motif and cannot bind calcium (Ishitani et al., 2000). The *GI* entry vector, *pENTR-1A-Amp-GI(s)* was given by Hong Gil Nam (DGIST, South Korea). All constructs used in this work are in Supplemental Table S2.

The plasmids for transformation of GFP-translational fusions to SOS3, SOS3-G2A and SOS3-C3A in Arabidopsis were produced using the pGreen system (Hellens et al., 2000). Plasmid pGreenII 0000 was used as backbone to generate the final construct. A 0.67 kb *Eco*RV fragment containing the CaMV35S expression cassette was ligated in the plasmid pGreenII 0000 cut with *Pvu*II. A 1.18 kb fragment *Eco*RV fragment containing the *nos:hygR* marker cassette was ligated into pGreenII 0000 cut with *Hpa*I. The yeast expression vector, pYES2GFP was digested with *Hind*III and *Xba*I to isolate a 0.81 kb band containing the multiple cloning sites (MCS) and the GFP coding sequence. The *Xba*I cutting site at the 3' end of this fragment was filled with Klenow to create a blunt end, and the purified MCS-GFP was ligated into the vector pGreenII 000 cut with *Hind*III and *Eco*RI (blunted) producing the final vector pGreenII 35S-GFP. The different alleles of SOS3 were generated by PCR using the forward primer: 5'-GGAAGCTTATGGGCTGCTCTGTATC (for SOS3 wild-type); 5'-GGAAGCTTATGGCCTGCTCTGTATC (for the Gly2 to Ala mutant); 5'-GGAAGCTTATGGGCGCCTCTGTATC (for the Cys3 to Ala mutant) and the reverse primer 5'-TCTGCGGCCGCGGAAGATACGTTTTGCAA using the SOS3 cDNA as template. *Hind*III and *Not*I restrictions sites are underlined in the primer sequence. The PCR fragments were digested with *Hind*III and *Not*I, purified and subcloned into the pGreenII vector cut with the same restriction enzymes.

For *sos3-1* complementation test, the different *SOS3* alleles were subcloned into the pBI321 vector (Martínez-Atienza et al., 2007). Plasmid *pYPGE-SOS3* (Guo et al., 2004) was digested with *Xba*I and *Xho*I to obtain a 0.67 kb fragment containing the wild-type SOS3 cDNA. Plasmid *pET-SOS3-G2A* (Ishitani et al., 2000) was digested with *Xba*I and *Xho*I to obtain a 0.69 kb fragment containing the mutant *SOS3-G2A* cDNA. The Cys3 to Ala (C3A) mutant of SOS3 was created by PCR using the forward primer: 5'- GATCTAGAATGGGCGCCTCTGTATC and the reverse primer 5'-AGCTCGAGGTTAGGAAGATACG. Primers add an *Xba*I site at the 5'end and an *Xho*I site at the 3' end of the amplified DNA. The *SOS3* alleles were ligated into the pBI321 vector cut with *Xba*I and *Xho*I.

The construct *proSOS3:SOS3-GFP* was used to mimic the expression of the native *SOS3* gene. For this, a translational fusion was constructed in which GFP was added to the C-terminus of a genomic

copy of *SOS3*. The expression of SOS3 was driven by its own promoter (2kb region upstream the ATG start codon). The *proSOS3:SOS3-GFP* construct was amplified and cloned into pGreenII as an *Xho*I-*Sph*I fragment. Forward and reverse oligonucleotides for cloning were SOS3 promoter: 5-CGGCATGCAGATCTAAAAAACAGGTAATGAGAATTTGG-3´ and fusion SOS3-GFP: 5´-CCACCTCGAGCGGAAGATACGTT-3´. Transgenic plants in the *sos3-1* background were selected using 15 mg/l of hygromycin in ½ MS plates. Homozygous plants from T2 generation were for tested for complementation of salt-sensitivity and used for further experimentation (Supplemental Figure S6). For SOS3 S-acylation test using transient expression in *N. benthamiana* and acyl resin-assisted capture, the wild-type allele of SOS3, the non-myristoylatable mutant SOS3-G2A, the non-palmitoylatable mutant SOS3-C3A and the non-myristoylatable, non-palmitoylatable double mutant SOS3-G2A/C3A were generated by PCR using the following forward primers: 5´-AAAAAGCAGGCTTCATGGGCTGCTCTGTATCGAAGAAG (for SOS3 WT); 5´-AAAAAGCAGGCTTCATGGCATGCTCTGTATCGAAGAAGAAG (for the G2A mutant); 5´-AAAAAGCAGGCTTCATGGGCGCATCTGTATCGAAGAAGAAG (for the C3A mutant); 5´-AAAAAGCAGGCTTCATGGCAGCATCTGTATCG AAGAAGAAGAAG (for the G2A and C3A double mutant), and the reverse primer 5´-AGAAAGCTGGGTCGGAAGATACGTTTTGCAATTCCATTTCT for all the above PCRs. Amplification products were subcloned using LR II clonase (Invitrogen) into the Gateway-compatible pYL436 vector, which confers C-terminal TAP tags (Rubio et al., 2005).

Transient expression in *Nicotiana benthamiana* leaves

Agrobacterium tumefaciens strain GV 3101 was transformed with constructs indicated in figure legends. *Agrobacterium* grew in LB media supplemented with 10 mM MES, 20 µM acetosyringone, and antibiotics dependent on the constructs and culture media were washed with infiltration solution (10 mM $MgCl_2$, 10 mM MES, and 100 µM acetosyringone). *Agrobacterium* transformed with P19, repressor of silencing was included (Lakatos et al., 2004). For co-infiltration, each of *Agrobacterium* cultures was OD_{600}0.5 in the final infiltration solution, which was infiltrated to leaves of three to four-week old *N. benthamiana* plants through stomata. Infiltrated *N. benthamiana* plants were incubated 2-3 days, and then treated if needed with 100 mM NaCl or 3 mM $CaCl_2$ solutions by infiltration into leaves in the morning (ZT2). After 10 h, salt-treated *N. benthamiana* leaves were harvested for further experiments such as co-IP or confocal laser fluorescence microscopy.

Bimolecular Fluorescence Complementation (BIFC) and Laser-Scanning Confocal Microscopy

Plasmid constructs for BiFC were transformed into *Agrobacterium tumefaciens* strain GV3101. Two days after *Agrobacterium* infiltration into *N. benthamiana* leaves, solutions of 100 mM NaCl or 3 mM $CaCl_2$, with or without 2 mM EGTA were infiltrated into epidermal cells, and 6-8 h later YFP signals were detected under confocal laser scanning microscope (FV 1000 Olympus). Excitation and emission wavelengths for YFP are 515 nm and 527 nm, respectively. The same settings were used for fluorescence detection in all the samples within the same experiment.

For GI-GFP fluorescence microscopy in Arabidopsis, 5-day old *35S:GI-GFP* and *35S:GFP* (GFP)

plants were treated with or without 100 mM NaCl for 8 h. GFP signals were detected under confocal laser scanning microscope (FV 100 Olympus) at excitation and emissions wavelengths of 488 nm and 516 nm, respectively.

For regular confocal microscopy after 2-bromo-palmitate treatment, plants were incubated for 10 min with 0.1% Triton and 0.2 µg/ml DAPI (Sigma), washed three times with ½ MS media and then images were taken. DAPI was exited with 405 laser and emission collected between 440-480 nm. GFP was exited with 488 laser and emission collected between 490-550 nm. Arabidopsis seedlings were imaged between slide and cover glass. The pictures were taken using a Zeiss LSM 780 with the objective 40x 1.1NA Water LD C-Apochromat Korr M27 (DIC). Image acquisitions were performed sequentially and analyzed using FIJI software, version 1.57. DAPI signal was used as a ROI to establish the localization of the nuclei to measure the fluorescence intensity (mean gray value (a.u.)) of *proSOS3::SOS3GFP* plants in different cellular compartments.

Co-localization analysis of the SOS3 and SOS3-C3A with the plasmamembrane LTI6b:RFP and the ER-mCherry CD3-959 markers (Nelson et al., 2007; Krebs et al., 2012) was performed using the Coloc2 plugin for Fiji. In the case of the ER images, Pearson's correlation coefficients were calculated using the ER-marker signal surrounding the nuclei as a ROI.

Analysis of SOS3 *S*-acylation *in planta* through acyl resin-assisted capture (acyl-RAC)

Agrobacterium cells transformed with the constructs of wild-type SOS3 and mutant SOS3 C-terminally tagged with TAP tag were infiltrated in *N. benthamiana* leaves, and wild-type and mutant SOS3 proteins were transiently expressed during 72 h. For each sample 1 g of leaf tissue was ground in liquid nitrogen and resuspended in 7.5 ml buffer containing 100 mM Tris-HCl (pH 7.5), 1% SDS, 1 mM PMSF, 1 mM EDTA and 30 mM N-ethylmaleimide. Leaf suspensions were first heated at 40°C for 10 min and then mixed gently for 1 h at 23°C. Debris was removed by centrifugation at 12000 *g* for 10 min at 23°C and supernatants were passed through 0.2 µm filters before precipitation with 85% acetone at -20°C for 1 h. Precipitated proteins were collected by centrifugation at 12000 *g* for 10 min at 4ºC. Pellets were washed once in 70% acetone and dried at 42°C for 30 min. Each protein pellet was dissolved in 20 ml binding-buffer consisting of 100 mM Tris-HCl (pH 7.5), 1% SDS, 1 mM EDTA and 4 M urea. Solutions were cleared by centrifugation at 12000 *g* for 10 min at 23°C and filtered through 0.2 µm filters. Fifty-µl aliquots were withdrawn from these solutions to be analyzed by immunoblot as "input". Sample solutions were divided in two 10-ml parts and hydroxylamine was added at 0.5 M final concentrations to one of these parts, whereas the other served as untreated control. Each sample solution was incubated with 200 µl thiopropyl-sepharose 6B resin (Sigma) overnight at 23°C under gentle mixing. The resin slurries were washed with 5 ml binding-buffer and covalently bound proteins were eluted in 200 µl of a buffer containing 20 mM Tris-HCl (pH 6.8), 0.4% SDS and 50 mM DTT.

RNA isolation and RT-qPCR

Total RNA (3 µg) extracted using RNeasy Plant Mini Kit (Qiagen) and treated with DNase (Sigma)

was used for synthesis of first-strand cDNA using first-strand cDNA using the ReverTra Ace-α-® (Toyobo Co. Ltd). Amplified products were detected using iQ™ SYBR® Green Supermix (Bio-Rad) in a thermal cycler (CFX384 C1000TM Real time system, Bio-Rad). The efficiency value of amplification for each primer set was checked by measuring the abundance of transcripts from cDNA dilutions according to the manufacture guidebook (real-time PCR applications guide, Bio-Rad). RT-qPCR conditions for *CO* and *FT* were as follows: 95°C for 5 min, 55 cycles of 95°C for 15 s, 55°C for 15 s and 72°C for 15 s, followed by 95°C for 10 s, 65°C for 5 s, and 95°C for 5 s for melting curves. RT-qPCR conditions for *GI* and *At5g12240* (internal control) were as follows: 95°C for 10 min, 45 cycles of 95°C for 10 s, 58°C for 30 s and 72°C for 10 s, followed by 65°C for 5 s and 90°C for 5 s for melting curves. *At5g12240* was used as a reference gene to normalize the transcript levels by the comparative cycle threshold (ΔΔCt) method (Livak and Schmittgen, 2001). Each data point shown is the average of independent amplifications from three biological replicas, each with three technical replicas. The raw data and statistical analysis of *CO*, *FT* and *GI* expression shown in Figure 1C are given in Supplemental Data Set S1 (available online). Primers used for RT-qPCR are in Supplemental Table S1. The list of constructs used in this study is in Supplemental Table S2.

Statistical analyses

Statistical analyses were performed with software Prism (GraphPad). All statistical analyses performed in this study can be found in Supplemental Data Set S1.

SUPPLEMENTAL FIGURES

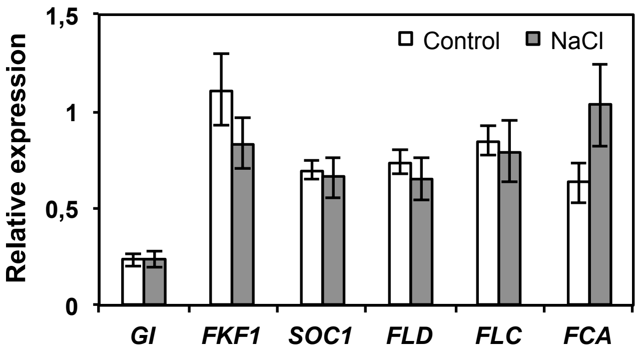

Supplemental Figure S1. Expression under salt stress of genes regulating flowering.

Supports Figure 1. Two-week old wild-type plants (Col-0 *gl1*) grown in long-day were treated with 100 mM NaCl at ZT0. Transcript levels of *GI, FKF1, FLD*, *SOC1*, *FLC* and *FCA* at ZT16 were measured by RT-qPCR. Bars represent means ± SEM from three technical replicates. Means of control and treated plants for each gene were not significantly different by Student's t-test at $p>0.1$. The experiment was repeated at least twice with similar results.

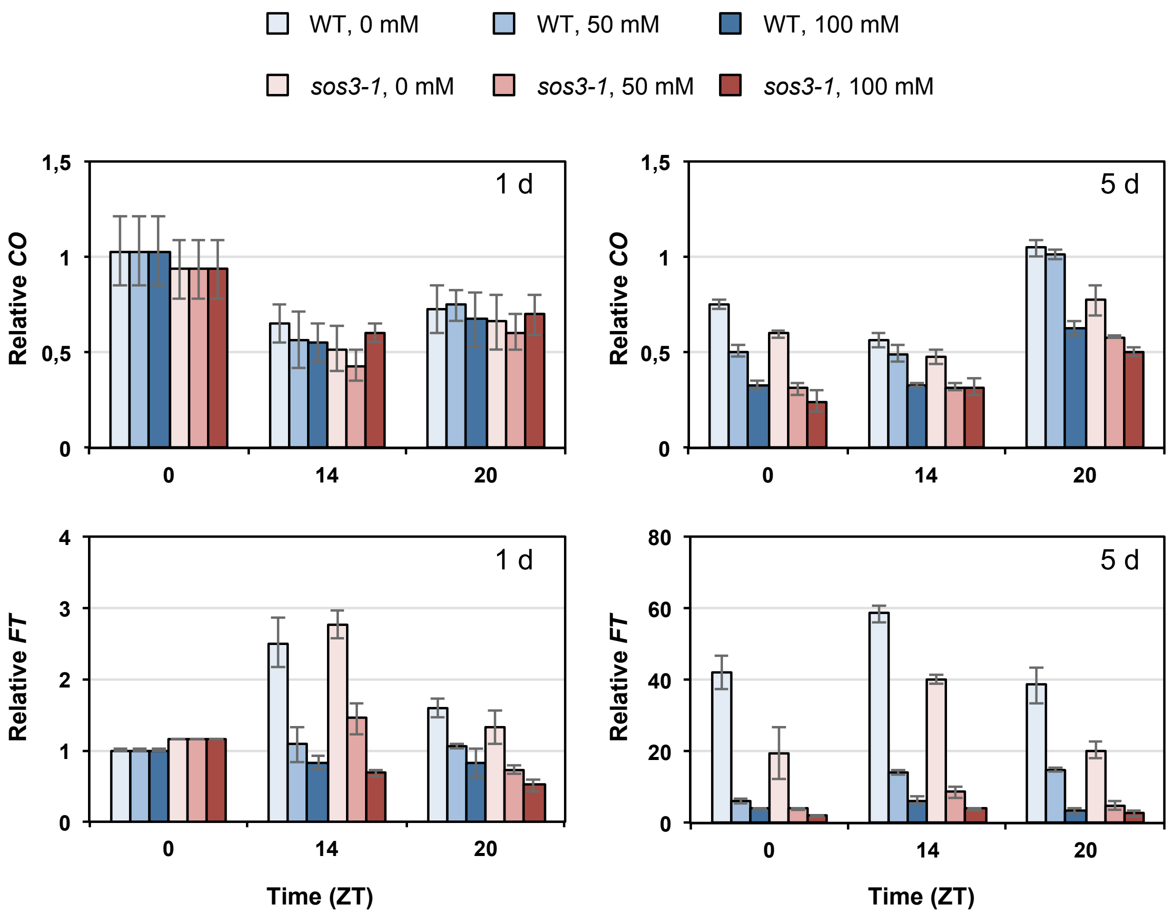

Supplemental Figure S2. Expression of *CO* and *FT* at 1- and 5-day under salt stress.

Supports Figure 1. Eight-day old plants of Col-0 *gl1* and *sos3-1* growing on ½ MS media (1% sucrose) under long-day conditions were treated with 100 mM NaCl at ZT0, and harvested at ZT14 and ZT20 of the same day (1 d), and at ZT0, ZT14, and ZT20 of the 5th day (5 d) after salt treatment. Transcript levels of *CO* and *FT* were measured by RT-qPCR and normalized to that of *At5g12240*. Error bars represent the SEM from three biological replicates, each with three technical replicates. A complete statistical analyses of these data can be found in Supplemental Table S1.

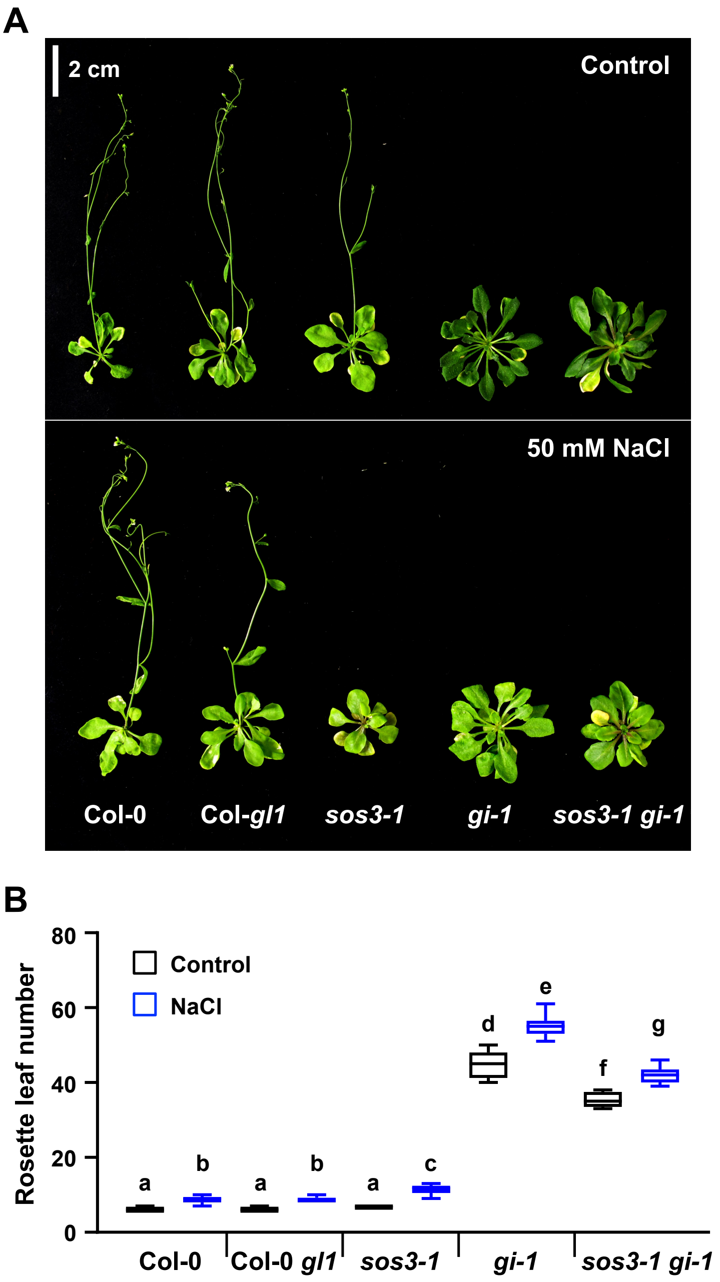

Supplemental Figure S3. Flowering phenotype of *sos3-1 gi-1* double mutant plants.

Supports Figure 1. **(A)** Effect of salt on the flowering time in wild-type (Col-0 and Col-0 *gl1*), *sos3-1*, *gi-1*, and *sos1-1 gi-1* double mutant. Ten-day old seedlings were transferred to MS media supplemented or not with 50 mM NaCl. The photographs were taken after bolding. Representative plants are shown. **(B)** Rosette leaf number at bolting time of plants grown with and without salt, as in (A), to score flowering time. Data is shown as dot plots, center lines show the medians. Monitored plants were Col-0 (control, n=15; NaCl, n=10), Col-0 *gl1* (n=13; n=10), *sos3-1* (n=15; n=13), *gi-1* (n=12; n=9), and *sos3-1 gi-1* (n=9; n=9). Statistical analysis was performed by one-way ANOVA followed by Fisher's LSD test; letters indicate significant differences at $p<0.05$.

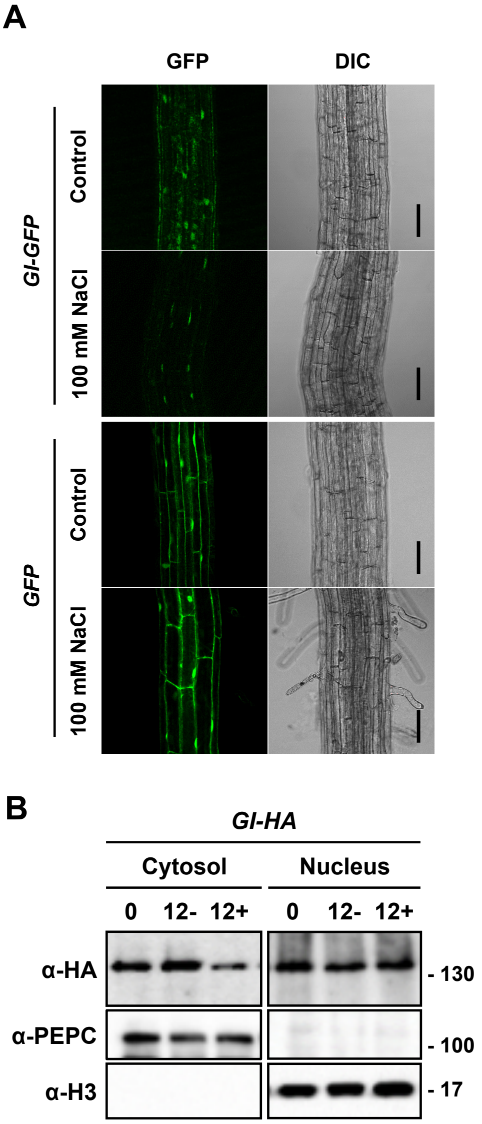

Supplemental Figure S4. Salt induced degradation of GI protein occurs in cytosol.

Supports Figure 2. (**A**) Five-day old *35S:GI-GFP* (GI-GFP) and *35S:GFP* (*GFP*) plants were treated with or without 100 mM NaCl for 8 h. Roots were visualized by differential interference contrast (DIC) and GFP signals were detected under confocal microscope. Bar represents 50 µm. (**B**) Two-week old plants of Arabidopsis overexpressing *GI-HA* were treated with (12+) or without (12-) 100 mM NaCl for 12 h. Cytosolic and nuclear proteins were extracted and submitted to immunoblotting with α-HA antibodies. α-PEPC and α-H3 antibodies were used for cytosolic and nuclear markers, respectively.

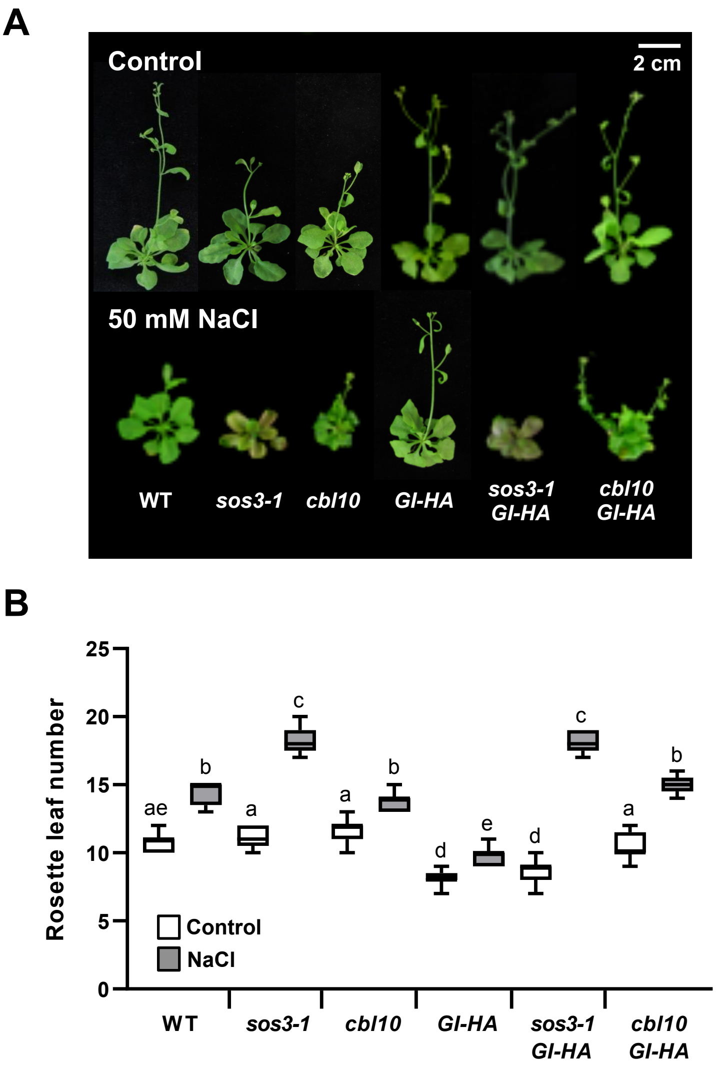

Supplemental Figure S5. CBL10 is not involved in the salt-induced late flowering.

Supports Figure 1. (**A**) Eight-day old plants of Col-0 (WT), *sos3-1, cbl10,* and the same genotypes over-expressing *GI* (*GI-HA, sos3-1 GI-HA, cbl10 GI-HA*) were transferred to MS media supplemented with 50 mM NaCl. Rosette leaf number was counted at bolting as flowering time. (**B**) Box plots of flowering time. Center lines show the medians; box limits indicate the 25th and 75th percentiles; whiskers extend to the minimum and maximum (n=9). Letters indicate significantly different means at $p<0.01$, Fisher's LSD test.

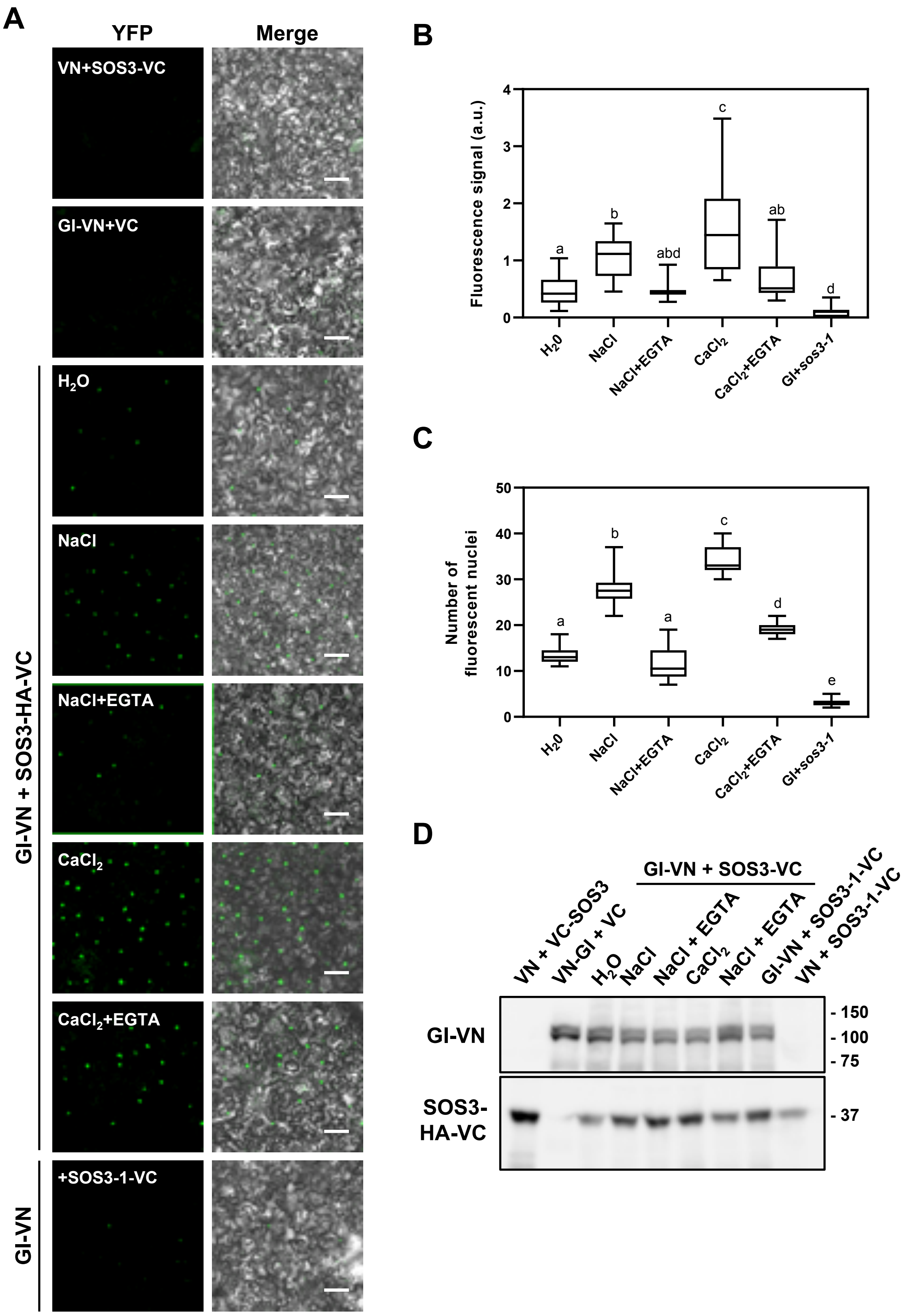

Supplemental Figure S6. BiFC of SOS3 and GI in *N. benthamiana* leaves.

Supplemental Figure S6. BiFC of SOS3 and GI in *N. benthamiana* leaves.

Supports Figure 3. **(A)** Tagged GI-VN and SOS3-HA-VC were transiently expressed in *N. benthamiana* leaves and plants were treated for 8 h with 100 mM NaCl, or 3 mM $CaCl_2$, with or without 2 mM EGTA. Water was used as mock treatment and the SOS3-1 mutant protein as the negative control. Fluorescent signals were detected under confocal laser scanning microscope. Bar represents 100 μm. (**B**) Quantitation of BiFC fluorescence shown in (A)**.** The fluorescence intensity was measured in frames of 0.4 mm^2 captured with x20 magnification objective. Data is shown as box plots. Letters indicate significantly different means, p <0.05 by Fisher's LSD test, n ≥ 3. (**C**) The number of fluorescent nuclei in five images (0.4 mm^2) of three biological replicas was counted and the results are shown as box plots: center lines show the medians; box limits indicate the 25th and 75th percentiles; whiskers extend to the minimum and maximum. Letters indicate significantly different means, p <0.001 by Fisher's LSD test, (n≥10); means with the same letter are statistically similar. (**D**) Expression and stability of proteins used for BiFC analyses. Immunoblots with total proteins from the *N. benthamiana* leaves used for the BiFC assay shown in (A). GI-VN and SOS3-HA-VC were detected with α-GFP and α-HA antibodies, respectively.

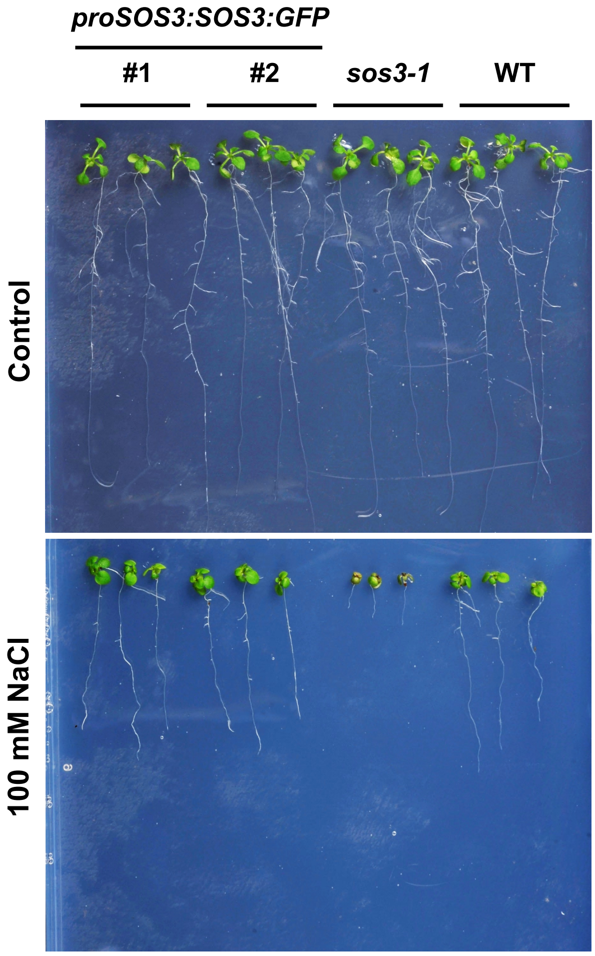

Supplemental Figure S7. Functional validation of the *proSOS3:SOS3:GFP* construct.

Supports Figure 4. Mutant *sos3-1* was transformed with construct *proSOS3:SOS3:GFP*. Seven-day old seedlings were transferred to vertical ½ MS plates with or without 100 mM NaCl and cultured for 1 week. Shown are two independent transgenic lines with the *proSOS3:SOS3:GFP* construct, compared to untransformed *sos3-1* mutant and the wild-type Col-0 *gl1*.

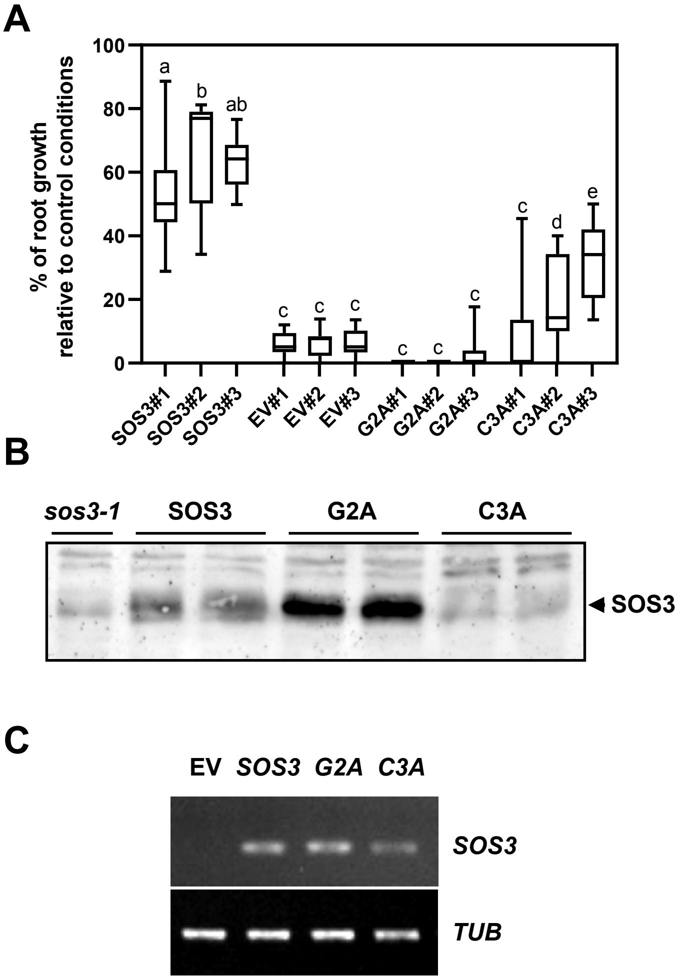

Supplemental Figure S8. Non-palmitoylated SOS3-C3A can partially rescue the salt sensitivity of *sos3-1*.

Supports Figure 5. (**A**) The *sos3-1* mutant was transformed to express cDNAs of wild-type SOS3 and mutant proteins G2A and C3A from the *35S* promoter. Transformants with empty vector (EV) were used as negative controls. Shown is the root growth of 7-day old seedlings of three independent transgenic lines for each transformation, 10 days after being transferred to vertical plates with MS supplemented with 100 mM NaCl. Growth data is relative to root growth of the same lines in plates without salt, as percent (n=9 seedlings per line and condition). Means followed by the same alphabet letter are not significantly different at p<0.05 by the Fisher's LSD test. (**B**) Immunoblot of whole seedling protein extracts probed with α-SOS3 polyclonal antibodies; two independent lines of each transformation of *sos3-1* transformed with the above constructs are shown, with the *sos3-1* mutant as control; the arrowhead indicates the SOS3 proteins. Note the low abundance of the SOS3-1 and SOS3-C3A proteins. **(C)** mRNA abundance of transgenes measured by RT-qPCR in 2-week old seedlings of representative transgenic lines selected for further research (lines SOS3#2, EV#1, GA2#3 and C3A#3). TUBULIN was used as loading control.

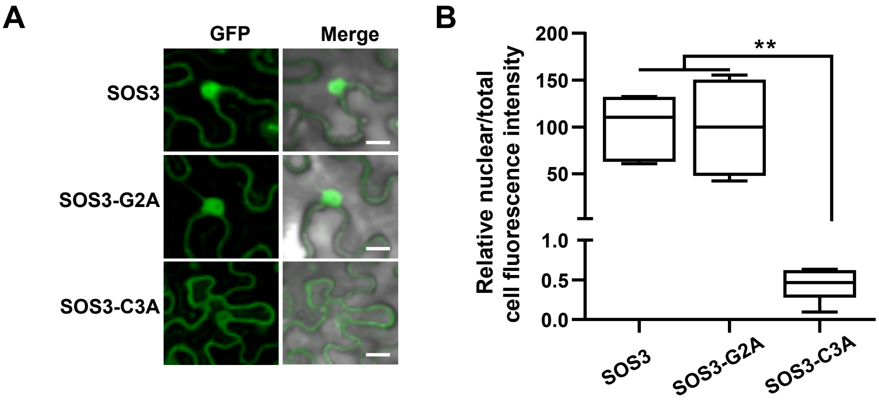

Supplemental Figure S9. Subcellular distribution of non-palmitoylated SOS3.

Supports Figures 5 and 6. (**A, B**) Mutation of the *S*-acylation site in SOS3 abrogates import into the nucleus. (**A**) Transient expression in *Nicotiana* of GFP-fused to the C-terminal part of SOS3, SOS3-G2A, and SOS3-C3A inspected under a confocal microscope. Scale bar represents 20 μm. (**B**) Normalized nuclear fluorescence intensity vs. total cell fluorescence. Shown in the Box plot: center lines show the medians; box limits indicate the 25th and 75th percentiles; whiskers extend to the minimum and maximum (n≥4). Asterisks indicate significantly different means of samples with and without salt for each genotype at p<0.01 by Fisher's LSD test.

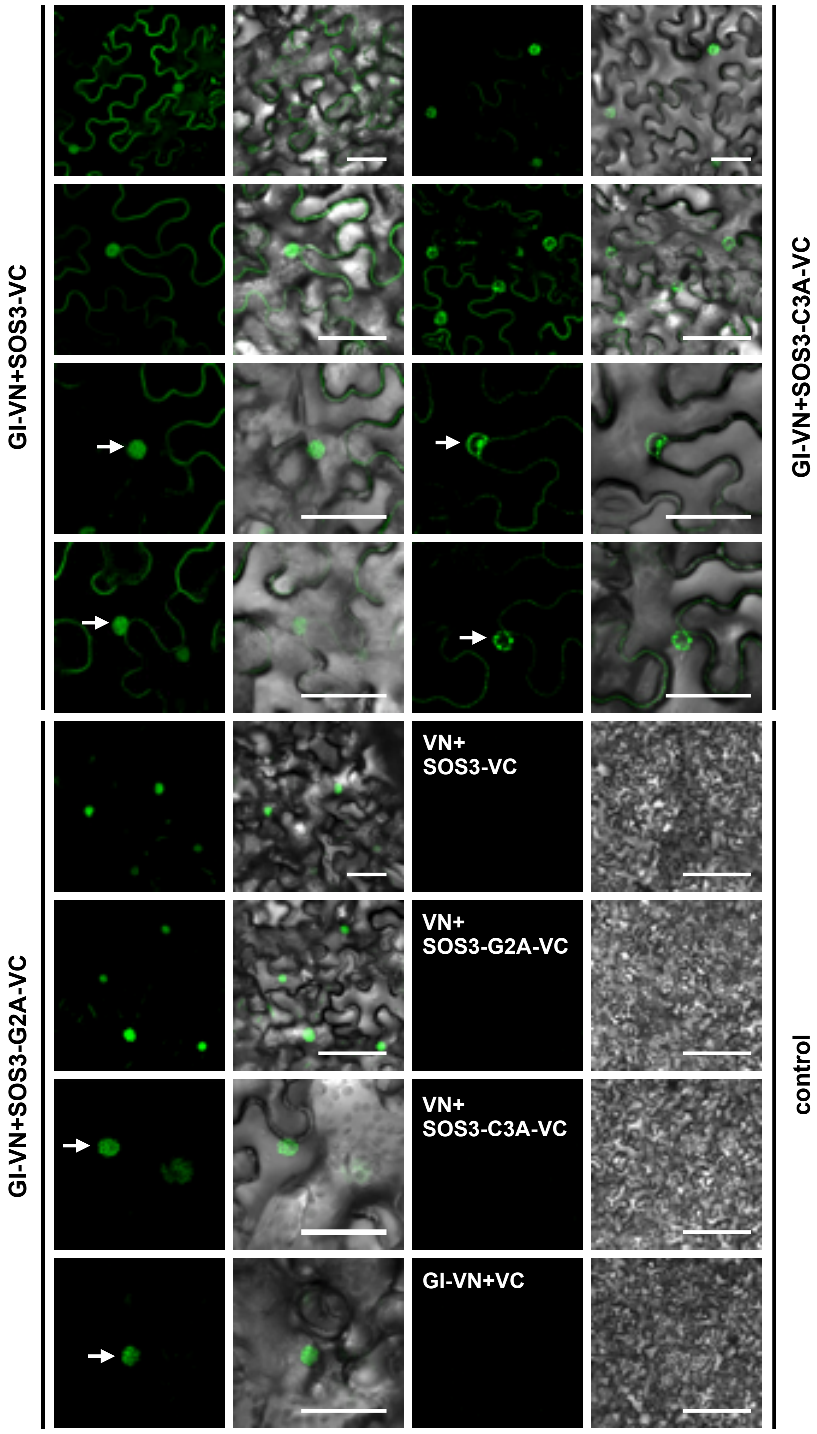

Supplemental Figure S10. Non-palmitoylated SOS3 fails to interact with GI in the nucleus. Supports Figure 6. *N. benthamiana* leaves were infiltrated with constructs as indicated. Fluorescent signals of GI and SOS3 interaction were detected under confocal microscope. Empty vectors were used as controls as indicated. Bars represent 50 μm.

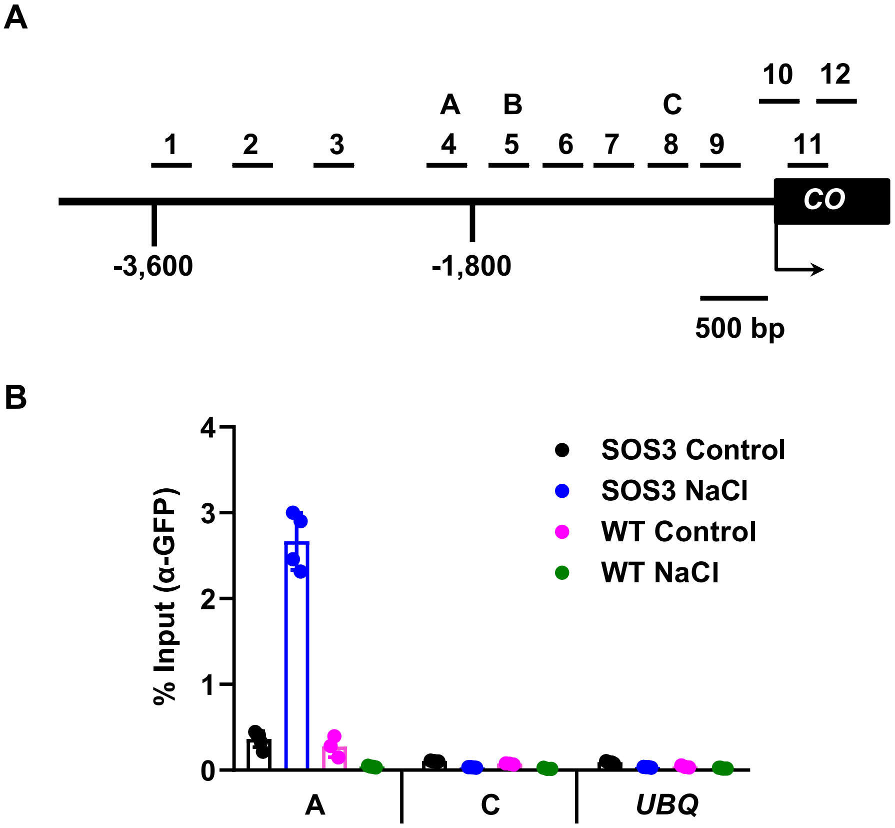

Supplemental Figure S11. SOS3 associates with the *CO* promoter.

Supports Figure 9. (**A**) Schematic drawing of the *CO* promoter and the amplicon locations (A, B, and C) for ChIP analysis, as defined by Sawa et al. (2007). The amplicon regions 4 and 8 in Sawa et al. (2007) are used as A-B and C, respectively, in this study. (**B**) Chromatin isolated from two-week-old *SOS3-GFPox* (SOS3) and wild-type Col-0 plants (WT; no GFP present) treated with or without 100 mM NaCl for 10 hours was immunoprecipitated with an α-GFP antibody. Immunoprecipitated and input DNA were used as templates for qPCR using primers specifically targeting to the amplicons A, C; *UBQ10* was used as control. Data is fragment enrichment as percent of input DNA. Error bar represents SEM (n≥3). Letters in fragment A indicate significantly different means at $p<0.01$, Fisher's LSD test. No statistical differences in fragment C and *UBQ*.

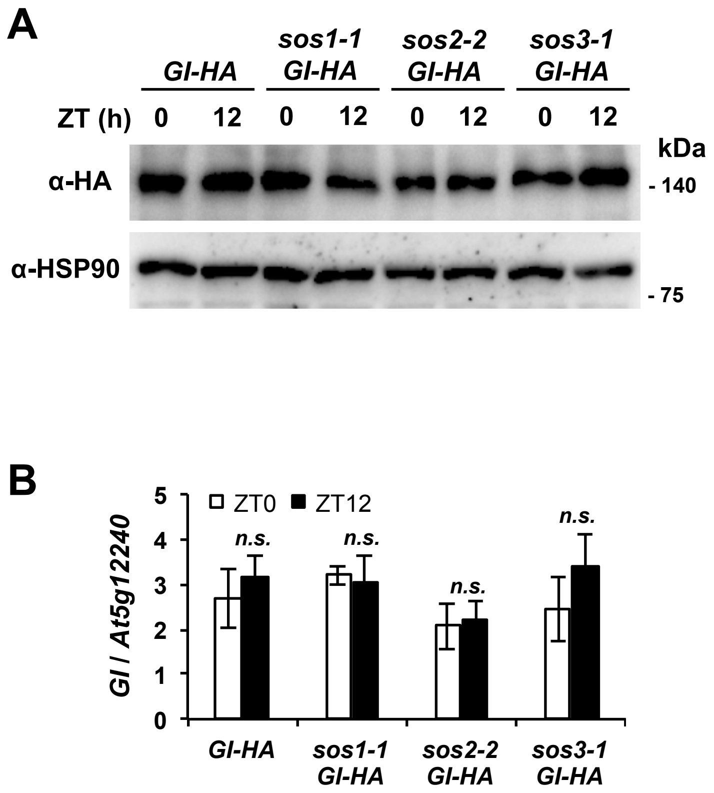

Supplemental Figure S12. Equal *GI* gene expression in *sos1,2,3* mutants.

(A) Total proteins were extracted from 2-week old *GI-HA*, *sos1-1 GI-HA*, *sos2-2 GI-HA* and *sos3-1 GI-HA* plants harvested at ZT0 and ZT12. Immunoblots with HA antibody was performed to detect GI protein. α-HSP90 antibody was used for a loading control. **(B)** Transcript levels of *GI-HA* were examined by RT-qPCR and normalized by the expression levels of At5G12240 as an internal control. Data represent means ± SEM, *n*=3, where *n* means the number of biological replicates. Significant differences are indicated (*n.s.*, not significant compared with ZT0 using a two-tailed Student's *t* test).

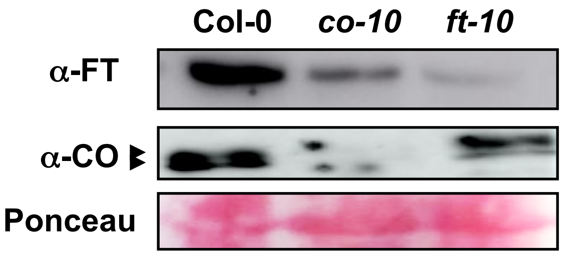

Supplemental Figure S13. Specificity of CO and FT antibodies.

Immunoblots sowing FT and CO protein presence in plant extracts from WT (Col-0) and knock-out mutants of *FT* (*ft-10*) and *CO* (*co-10*). Fifty µg of total protein were loaded per lane and incubated with α-FT(upper panel) and α-CO (middle panel). Lower panel shows Ponceau staining as loading control. Double band of CO is marked by arrowheads.

Supplemental Table S1. List of primers used in this study and their purpose.

Primers	Sequence	Reference	Purpose
attB1 sequence	AAAAAGCAGGCTNN	Invitrogen	Gateway
attB2 sequence	AGAAAGCTGGGTN	Invitrogen	
attB1 SOS3-F	AAAAAGCAGGCTTCATGGGCTGCTCTGTATCGAA	This study	Plasmid construction
attB1SOS3-G2A-F	AAAAAGCAGGCTTCATGGCCTGCTCTGTATCG	This study	
attB1SOS3-C3A-F	AAAAAGCAGGCTTCATGGGCGCCTCTGTATCGAAG A	This study	
attB1-SOS3-K/A F	AAAAAGCAGGCTTCATGGGCTGCTCTGTATCG	This study	
attB2-SOS3-R/No Stop	AGAAAGCTGGGTCGGAAGATACGTTTTGCAAT	This study	
attB1 SOS2-F	AAAAAGCAGGCTTCATGACAAAGAAAATGAGAAG	Kim et al., 2013	
attB2-SOS2-R/Stop	AGAAAGCTGGGTTCAAAACGTGATTGTTCTGAG	Kim et al., 2013	
attB2-SOS2-R/No Stop	AGAAAGCTGGGTCAAACGTGATTGTTCTGAGAAT	Kim et al., 2013	
attB1-FKF1-F	AAAAAGCAGGCTTCATGGCGAGAGAACATGCGAT	This study	
attB2-FKF1-R/Stop	AGAAAGCTGGGTCTTACAGATCCGAGTCTTGCC	This study	
FwPr-SOS3-GFP	CGGCATGCAGATCTAAAAAACAGGTAATGAGAATTT GG	This study	
RvPr-SOS3-GFP	CCACCTCGAGCGGAAGATACGTT	This study	
UBQ-qRT-F	GACGCTTCATCTCGTCC	This study	qPCR
UBQ-qRT-R	GTAAACGTAGGTGAGTCC	This study	
CO-qRT-F	ATTCTGCAAACCCACTTGCT	Kim et al., 2013	
CO-qRT-R	CCTCCTTGGCATCCTTATCA	Kim et al., 2013	
FT-qRT-F	CTGGAACAACCTTTGGCAAT	Kim et al., 2013	
FT-qRT-R	AGCCACTCTCCCTCTGACAA	Kim et al., 2013	
GI-qRT-F	CTAGTTGCTGGCCTTCCAGC	This study	
GI-qRT-R	TACAGCAGCATCAAGTGCATCT	This study	
AT5G12240-qRT-F	TCTCGAAAGCCTTGCAAAATCT	Khaleda et al., 2017	
AT5G12240-qRT-R	AGCGGCTGCTGAGAAGAAAGT	Khaleda et al., 2017	
SOC1-qRT-F	AACAACTCGAAGCTTCTAAACGTAA	This study	
SOC1-qRT-R	CCTCGATTGAGCATGTTCCT	This study	
FLC-qRT-F	GCTACTTGAACTTGTGGATAGCAA	This study	

Supplemental Table S1. List of primers used in this study and their purpose (continued).

Primers	Sequence	Reference	Purpose
FLC-qRT-R	GGAGAGGGCAGTCTCAAGGT	This study	qPCR
FKF1-qRT-F	GTTGTACCGCCTCCAAGACT	This study	
FKF1-qRT-R	AGATGATGACCCTACCACACG	This study	
FLD-qRT-F	GAAAGTGTGGGAGATGGAAGG	This study	
FLD-qRT-R	CAGATTGAGCCATGTTTGCAG	This study	
FCA-qRT-F	CAAGGACAAGAGAACTGGACAG	This study	
FCA-qRT-R	ATCGAACTTGAACAGGACCAG	This study	
CO_pro_amp_#4(A)_LP	TATGGTCCCTCGACTCTTATTCTCT	Sawa et al., 2007	ChIP
CO_pro_amp_#4(A)_RP	GCCTTCGGATAACTGTTACGAGTAA	Sawa et al., 2007	
CO_pro_amp_#5(B)_LP	ATTTACTCTTCATGAACTCGAACCA	Sawa et al., 2007	
CO_pro_amp_#5(B)_RP	ACTGGTTTTACGATGAATGTAATGG	Sawa et al., 2007	
CO_pro_amp_#8(C)_LP	TGGTTACCAAGTGCAAATTTCTACA	Sawa et al., 2007	
CO_pro_amp_#8(C)_RP	GAGAATCATATCGGAAAAGTGACATGAA	Sawa et al., 2007	
UBQ10_For_LP	TCCAGGACAAGGAGGTATTCCTCCG	Sawa et al., 2007	
UBQ10_Rev_RP	CCACCAAAGTTTTACATGAAACGAA	Sawa et al., 2007	

Supplemental Table S2. List of constructs used in this study.

**Name**	**Construct**	**Vector name**	**Reference**	**Purpose**
GI-VN	*35S:GI-VN*	pDEST-^{GW}VYNE	Kim et al., 2013	BIFC
VC-SOS2	*35S:VC-SOS2*	pDEST-VYCE(R)^{GW}	Kim et al., 2013	
SOS3-VC	*35S: SOS3-VC*	pDEST- ^{GW}VYCE	This study	
SOS3-1-VC	*35S:SOS3-1-VC*	pDEST-^{GW}VYCE	This study	
SOS3-G2A-VC	*35S:SOS3-G2A-VC*	pDEST- ^{GW}VYCE	This study	
SOS3-C3A-VC	*35S:SOS3-C3A-VC*	pDEST- ^{GW}VYCE	This study	
VC-FKF1	*35S:VC-FKF1*	pDEST-VYCE(R)^{GW}	This study	
GI-HA	*35S:GI-HA*	pGWB14	Kim et al., 2013	Transient expression
GI-GFP	*35S:GI-GFP*	pK7WGF	Kim et al., 2013	
SOS3-MYC	*35S:SOS3-MYC*	pCambia1300PT	Kim et al., 2013	
SOS3-FLAG	*35S:SOS3-FLAG*	pGWB11	This study	
SOS3-GFP	*35S:SOS3-GFP*	Modified pGreen0000	This study	
SOS3-G2A-GFP	*35S:SOS3-G2A-GFP*	Modified pGreen0000	This study	
SOS3-C3A-GFP	*35S:SOS3-C3A-GFP*	Modified pGreen0000	This study	
MYC-SOS3-1	*35S:MYC-SOS3-1*	pEarleygate203	This study	
MYC-FKF1	*35S:MYC-FKF1*	pEarleygate203	This study	
SOS3-TAP	*2x35S:SOS3-TAP*	pYL436	This study	
SOS3-G2A-TAP	*2x35S:SOS3-G2A-TAP*	pYL436	This study	
SOS3-C3A-TAP	*2x35S:SOS3-C3A-TAP*	pYL436	This study	
SOS3-G2AC3A-TAP	*2x35S:SOS3-G2A C3A-TAP*	pYL436	This study	
SOS3	*35S:SOS3*	pBI321	This study	Transgenic plants
SOS3-G2A	*35S:SOS3-G2A*	pBI321	This study	
SOS3-C3A	*35S:SOS3-C3A*	pBI321	This study	
proSOS3:SOS3-GFP	*proSOS3:SOS3-GFP*	pGreenII	This study	

SUPPLEMENTAL REFERENCES

Guo, Y., Qiu, Q.-S., Quintero, F.J., Pardo, J.M., Ohta, M., Zhang, C., Schumaker, K.S., and Zhu, J.-K. (2004). Transgenic evaluation of activated mutant alleles of SOS2 reveals a critical requirement for its kinase activity and C-terminal regulatory domain for salt tolerance in Arabidopsis thaliana. The Plant Cell **16,** 435-449.

Hellens, R.P., Edwards, E.A., Leyland, N.R., Bean, S., and Mullineaux, P.M. (2000). pGreen: a versatile and flexible binary Ti vector for Agrobacterium-mediated plant transformation. Plant Mol Biol **42,** 819-832.

Ishitani, M., Liu, J., Halfter, U., Kim, C.-S., Shi, W., and Zhu, J.-K. (2000). SOS3 function in plant salt tolerance requires N-myristoylation and calcium binding. The Plant Cell Online **12,** 1667-1677.

Kim, W.-Y., Ali, Z., Park, H.J., Park, S.J., Cha, J.-Y., Perez-Hormaeche, J., Quintero, F.J., Shin, G., Kim, M.R., Qiang, Z., Ning, L., Park, H.C., Lee, S.Y., Bressan, R.A., Pardo, J.M., Bohnert, H.J., and Yun, D.-J. (2013a). Release of SOS2 kinase from sequestration with GIGANTEA determines salt tolerance in Arabidopsis. Nat Commun **4,** 1352.

Krebs, M., Held, K., Binder, A., Hashimoto, K., Den Herder, G., Parniske, M., Kudla, J., and Schumacher, K. (2012). FRET-based genetically encoded sensors allow high-resolution live cell imaging of Ca^{2+} dynamics. Plant J **69,** 181-192.

Lakatos, L., Szittya, G., Silhavy, D., and Burgyán, J. (2004). Molecular mechanism of RNA silencing suppression mediated by p19 protein of tombusviruses. EMBO J **23,** 876-884.

Livak, K.J., and Schmittgen, T.D. (2001). Analysis of relative gene expression data using real-time quantitative PCR and the 2(-Delta Delta C(T)) Method. Methods **25,** 402-408.

Martínez-Atienza, J., Jiang, X., Garciadeblas, B., Mendoza, I., Zhu, J.-K., Pardo, J.M., and Quintero, F.J. (2007). Conservation of the salt overly sensitive pathway in rice. Plant Physiology **143,** 1001-1012.

Nelson, B.K., Cai, X., and Nebenführ, A. (2007). A multicolored set of in vivo organelle markers for co-localization studies in Arabidopsis and other plants. Plant J **51,** 1126-1136.

Rubio, V., Shen, Y., Saijo, Y., Liu, Y., Gusmaroli, G., Dinesh-Kumar, S.P., and Deng, X.W. (2005). An alternative tandem affinity purification strategy applied to Arabidopsis protein complex isolation: Alternative tandem affinity purification in Arabidopsis. The Plant Journal **41,** 767-778.

Sawa, M., Nusinow, D.A., Kay, S.A., and Imaizumi, T. (2007). FKF1 and GIGANTEA complex formation is required for day-length measurement in Arabidopsis. Science **318,** 261-265.
