# Supplementary material for: S-acylated and nucleus-localized SALT OVERLY SENSITIVE3/CALCINEURIN B-LIKE4 stabilizes GIGANTEA to regulate Arabidopsis flowering time under salt stress
Source: Plant Cell. 2022 Sep 22;35(1):298–317. doi: 10.1093/plcell/koac289 (PMC9806564; doi:10.1093/plcell/koac289)
Supplement: koac289_Supplementary_Data [file tpc22.00153PeerReviewReport.pdf › SupplementaryFile/tpc22.00153PeerReviewReport.pdf]

S-acylated and nucleus-localized SALT OVERLY SENSITIVE3/ CALCINEURIN B-LIKE4 stabilizes GIGANTEA to regulate Arabidopsis flowering time under salt stress

Hee Jin Park, Francisco Gamez-Arjona, Marika Lindahl, Rashid Aman, Irene Villalta, Joon-Yung Cha, Raul Carranco, Chae Jin Lim, Elena García, Ray A. Bressan, Sang Yeol Lee, Federico Valverde, Clara Sánchez-Rodríguez, Jose M Pardo, Woe-Yeon Kim, Francisco J. Quintero, and Dae-Jin Yun

Corresponding authors: Dae-Jin Yun, djyun@konkuk.ac.kr; Francisco J. Quintero, fjquintero@ibvf.csic.es; Woe-Yeon Kim, kim1312@gnu.ac.kr

Review timeline:

**TPC2021-RA-00153**	Submission received:	Mar. 1, 2022
	1st Decision:	Mar. 31, 2022 *revision requested*
**TPC2021-RA-00153R1**	1st Revision received:	July 19, 2022
	2nd Decision:	Aug. 24, 2022 *accept with minor revision*
**TPC2021-RA-00153R2**	2nd Revision received:	Aug. 29, 2022
	3rd Decision:	Sept. 1, 2022 *acceptance pending, sent to sci. ed*
	Final acceptance:	Sept. 16, 2022

REPORT:(The report shows the major requests for revision and author responses. Minor comments for revision and miscellaneous correspondence are not included. The original format may not be reflected in this compilation, but the reviewer comments and author responses are not edited, except to correct minor typographical or spelling errors that could be a source of ambiguity.)

TPC2021-RA-00153 1st Editorial decision – *revision requested* Mar. 31, 2022

We have received reviews of your manuscript entitled "S-acylated and Nuclear-Localized SOS3/CBL4 Stabilizes GIGANTEA to Regulate Arabidopsis Flowering Time under Saline Stress." Thank you for submitting your best work to The Plant Cell. The editorial board agrees that the work you describe is substantive, falls within the scope of the journal, and may become acceptable for publication, pending revision and potential re-review.

We ask you to pay more attention to the following points in preparing your revision: 1. Please add more alleles of mutants and independent transgenic lines for phenotypic analysis; 2. The flowering phenotype of *gi sos3* double mutants should be provided; 3. Please quantify the GI protein in the nucleus in GI-GFP variant lines.

-- Reviewer comments:

[Reviewer comments shown below along with author responses]

TPC2021-RA-00153R1 1st Revision received July 19, 2022

Reviewer comments and **author responses**:

Editorial comments:

We ask you to pay more attention to the following points in preparing your revision: 1. Please add more alleles of mutants and independent transgenic lines for phenotypic analysis; 2. The flowering phenotype of *gi sos3* double mutants should be provided; 3. Please quantify the GI protein in the nucleus in GI-GFP variant lines.

Q1. As we explain to Reviewer #3 (Q2), one representative line was used when the transgenic/mutant had been described elsewhere and phenotype consistency assessed at the time of production (e.g. lines GI-GFP, GI-NLS and GI-NES in Fig. 2) (Kim et al., 2013b). The *sos1,2,3* mutant lines expressing GI-HA (Fig 1 & 2) where obtained by genetic crossing, meaning that all lines shared the same transgenic event, which again was previous characterized and published (Kim et al., 2013a). We believe this is standard procedure with published biological resources, and proper references to these published materials are given in the manuscript. On the other hand, transgenic/complemented lines produced specifically for this work were selected as representative lines after comparing several independent lines representing unique transformation events (e.g. *sos3-1* complemented lines with SOS3 protein variants; see Supplemental Fig S7), and the *pSOS3:SOS3-GFP/sos3-1* line used for spinning disc confocal microscopy (see Supplemental Fig S6).

Q2. As requested, we have measured the flowering time of the double mutant *sos3-1 gi-1*. The result, presented in new Supplemental Figure S3, shows that the severe flowering time delay of the *gi-1* mutant is not aggravated in the *sos3-1 gi-1* double mutant, meaning that SOS3 effect on flowering time under saline stress in GI dependent. However, the further delayed flowering transition of these two mutant lines under salinity suggests that other positive flowering regulators besides GI are likely affected by salinity stress (see lines 165-178).

Q3. The GI-GFP lines tagged with and without NLS and NES had been produced and characterized extensively in a previous report (Kim et al., 2013b). Measurement of the nucleo-cytoplasmatic partition of these recombinant protein variants showed that spatial segregation was not all-or-none. The differences in flowering time measured here are fully coherent with the relative abundance of GI within the nucleus in these lines. Please see our reply to Q4 of Reviewer #2 for a complete answer to this specific concern.

Besides these additions, we have incorporated to the revised Ms a new western blot of CO and FT proteins including controls for antibody specificity, and measured the flowering time data of mutant *fkf1* as requested by Reviewer 2.

We believe that these explanations and the new data incorporated to the revised manuscript fulfill satisfactorily the Editorial and Reviewer's requests. We also understand that this exciting report brings about novel concepts regarding *S*-acylation dependent nuclear partitioning of proteins, and that the stress- and calcium-responsive protein SOS3 controls GI stability and becomes incorporated to *CO* chromatin to adjust flowering time upon an environmental insult. These groundbreaking new findings inevitably raise some more questions that, in turn, demand more research. However, not all answers can be provided and every derivative analyzed in one single piece of work.

<u>Reviewer #1:</u>

General assessment: Overall, this is an exciting story that exhibited how plants control diverse stress responses by spatial regulation of core signal components. I think the manuscript could be accepted after the following questions are resolved.

Thank you. Authors truly appreciate the encouraging assessment and the comments that have contributed to make this report stronger.

Major concerns:

Q1) The authors analyzed CO and FT expression in WT and *sos3* mutant under salt stress (Figure 1C). They performed technical repeats but not biological repeats, which renders this result less solid. I suggest the authors perform biological repeats and show the typical result for CO and FT expression.

Sorry, there was a typo in the original legend, which read: *three replicates with three technical replicates each*. The correct sentence is: *three <u>biological</u> replicates (plants) with three technical repeats each.* The mistake has been corrected (lines 632-635).

Q2) Palmitoylation and depalmitoylation regulate plasma membrane-nuclear shuttling of many proteins. Since the authors reported a palmitoylation regulated nuclear import, the traditional depalmitoylation regulated nuclear accumulation should be discussed.

Thank you for the advice. We have incorporated two examples of de-palmitoylation dependent nuclear import to illustrate the classical process, and amended the text to highlight the crucial difference with the novel mechanism we describe here (marked text in lines 419-430).

Minor concerns:

Q3) lane 97-101: not clear. It is not clear about the positive and negative regulators and how photoreceptors affect CO stability.

We have expanded the background information on *CO* gene expression and protein stability. Please see marked text in Introduction (lines 46-70).

Q4) lane 200-202: not accurate. It seems that overexpression of GI-HA in sos1 but not sos2 promoted early flowering under salt stress, and overexpression of GI-HA in sos2 but not sos1 promoted early flowering under normal conditions.

The sentence was correct in essence (overexpression of GI-HA produced earlier flowering in WT, *sos1* and *sos2* but not in *sos3*), albeit it might have not been sufficiently precise.

To make the statistical analysis more comprehensive we have amended Fig 1B to show all statistical categories at *p*<0.01 by Fisher's LSD test (see below). The apparent difference between *sos1/GI-HA* and *sos2/GI-HA* under normal conditions that the Reviewer refers to was not statistically significant; the different letter-tagging of these samples in the previous version of plot Fig 1B was a typographical error. In the corrected plot *sos1/GI*, *sos2/GI* and *sos3/GI* are category 'f' in control conditions. By contrast, *sos1/GI*, *sos2/GI* under salt stress are both category 'i' under salt stress whereas *sos3/GI* plants showed a much delayed flowering that was identical to that of the *sos3* mutant in the same conditions (both the only data in category 'c'). This implies that the *sos3* mutant background had enhanced flowering delay under saline stress that could not be corrected by GI overexpression. Moreover, for the revised manuscript we show that the severe flowering time delay of the *gi-1* mutant was not aggravated in the *sos3-1 gi-1* double mutant (new Supplemental Figure S3).

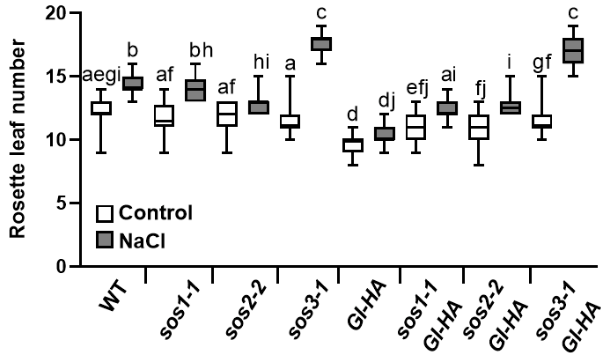

One 'anomaly' is that GI overexpression in the WT background produced early flowering under control conditions that is different (category 'd') to that of *sos1/GI*, *sos2/GI* and *sos3/GI* (category 'f'). This could be due to the capacity of GI to interact with all three SOS proteins. We have shown previously that GI forms a complex with SOS2 (Kim et al., 2013a), and we report here that GI also interacts with SOS3. In another work (provisional acceptance in *P.N.A.S.*) we report that GI physically interacts with SOS1, and that this last interaction results in altered circadian clock under salt stress. How the loss of these SOS-GI interactions in the *sos1/2/3* mutants, which are retained in the WT, affect GI function in setting flowering time under regular non- saline conditions will require additional research that, we think, is not the focus of this work. In this paper we aimed to provide a molecular basis for the critical observation of extra-delayed flowering time in the *sos3* mutant in a saline environment.

On the other hand, the later flowering time of *sos1/GI* and *sos2/GI* compared to that *WT/GI (GI-HA)* under saline

conditions is likely due to the greater intensity of the salt stress in these *sos* mutants, which could result in a faster rate of GI protein degradation compared to the *WT/GI* line. We have incorporated this explanation to the revised text (lines 165-178).

Q5) lane 263, need more background information. "Unlike thioester bonds, the covalent attachment of NEM to cysteine side groups is not sensitive to hydroxylamine."

Sorry, but we couldn't match this sentence with the main text and supplemental files. Nevertheless, we have improved the explanation of the biochemical basis of differential modifications of cysteines in lines 233-238.

Q6) lane 366 and 368, figure 7C should be "figure 7B". Corrected.

Q7) lane 447, not correct. "whereas the non-myristoylatable mutant SOS3-G2A could not yet flowered normally."

Corrected to 'However, SOS3-C3A was able to partially suppress the salt-sensitivity of mutant sos3-1, whereas the non-myristoylatable mutant SOS3-G2A could not restore salt tolerance but supported normal flowering.' (lines 407-409).

Reviewer #2:

Some major comments:

Q1) I believe the phenotype of *sos3* under salt stress is convincing (Fig 1A/B). In contrast, expression studies in Fig 1C are harder to interpret. FT levels appear to decrease in response to treatment, but the significance of the genotype by treatment term is not stated. Similarly, *CO* transcript analysis should include multivariate statistics. Moreover, *CO* transcript accumulation reaches a minimum in *sos3* + NaCl at ZT20 (i.e. at night, when CO is usually degraded). Is this physiologically relevant?

Diurnal oscillations of CO and FT transcript/protein abundance are influenced by numerous environmental inputs that fine-tune flowering time. Consequently, the interpretation of specific changes in individual elements acting in the interlocking transcriptional feedback loops controlling the developmental transition to flowering is not always straightforward. The Reviewer is right that the CO protein degrades at night due to the action of COP1 whereas *CO* transcription reaches maximal expression, as inhibition by CDFs is not exerted during the night period in LDs. This is the nature of the circadian/photoperiodic transcription-translation loop in wild-type Arabidopsis plants; *CO* transcription peaks at night while the CO protein gets degraded. This is consolidated knowledge. Because GI is a positive effector of *CO* transcription, it makes sense that the enhanced GI protein degradation in the *sos3-1* mutant under salt stress results in reduced transcription of *CO* and *FT* (Fig 1C), and in a greater flowering retardation compared to the WT (Fig 1B). Why the effects of salinity on *CO* transcription are more intense at night? Because plants use the circadian clock to anticipate diurnal changes, we can speculate that the transcription of *CO* at night, peaking right before dawn in the wild-type under saline stress (Fig 1C), will influence the abundance of the CO protein during daylight. This is the part of the stress response that is missing in the *sos3-1* mutant, in which *CO* transcription falls at night. Ultimately, the key factors in setting the flowering time is CO and FT protein abundance and we show in Fig 1D the enhanced decay of CO and FT proteins in salt-treated *sos3-1* mutant plants, in line with the greater flowering delay.

As requested, we have performed two-way ANOVA for factors 'time', 'genotype' and 'treatment'. As expected, the factors time, genotype and treatment were highly significant for *CO* and *FT*, whereas for *GI* the only significant factor was time of the day. Results are given in the main text (ln 151-159). Moreover, we provide the statistical analyses of genotypes and treatments at each time point in new Supplemental Table S1.

Q2) Addition of immunoblots of CO and FT as in Fig 1D is a nice idea. Unfortunately, this experiment lacks key controls (e.g. *co* and *ft* KO mutants) that could help verify antibody specificity.

We have produced an improved western blot, shown in new Fig 1D, that includes two loading controls. Moreover, negative controls with *co* and *ft* mutant plants to demonstrate antibody specificity have been incorporated as new Supplemental Figure S13. The replicated results are coherent with the previous data and show enhanced CO and FT protein decay in salt-treated *sos3-1* mutant plants.

Q3) There is an assumption throughout the text that SOS3 acts via GI. While plausible, the authors must show the phenotype of *gi sos3* double mutants to demonstrate epistasis between *gi* and *sos3*. Moreover, what is the flowering phenotype of *co* and *fkf1* mutants under salt stress?

We had demonstrated previously that the molecular basis for the salt-induced delay in flowering time is GI degradation (Kim et al., 2013a). To that basic concept we add now the finding that GI stability is differentially regulated in nuclear and cytosolic compartments, and that nuclear localized SOS3 stabilizes the GI nuclear pool. Therefore, we conclude that SOS3 acts in the photoperiodic flowering-time signaling via GI. As requested, we have measured the flowering time of the double mutant *sos3- 1 gi-1* in control and salt conditions. The results, incorporated to new Supplemental Figure S3, show that mutations *gi-1* and *sos3-1* are not additive regarding flowering time in both control and in saline conditions, meaning that SOS3 effect on flowering time under saline stress in GI dependent. However, the further delayed flowering transition of *gi-1* and *gi-1 sos3-1* mutant lines under salinity suggests that other positive flowering regulators are likely affected by salinity stress; FKF1 appears to be one of these factors (see new Fig. 8).

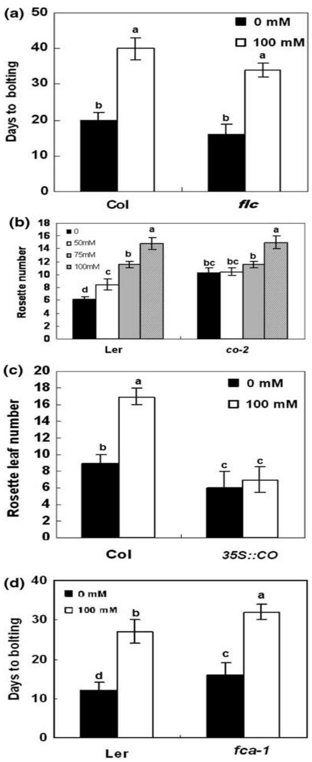

We have also included in Figure 8 the flowering time data requested for the *fkf1* mutant. As expected from its shared role with GI in photoperiodic flowering, *fkf1* plants have late flowering that is only slightly delayed further by salt (*p*=0.20; *t*-Test; n=6 plants).

The flowering time of *co* mutant plants in saline conditions has been published elsewhere (Li et al., 2007) (see below). In control conditions the *co-2* mutant displayed late flowering. Salinity delayed flowering in a dose response manner both in wild-type and *co-2* mutant plants, with a greater progression in the wild-type so that both genotypes reached a similar retardation at 100 mM NaCl. We explain this in lines 375- 378.

Fig. 6. Floral transition of the related mutant plants. (a) Flowering of *flc* mutant plants under 0 and 100 mM NaCl (mean ± SD; n = 40 plants each treatment). (b–c) Analysis of the flowering time of *co-2* (b) and 35S::CO (c) in response to salt treatments, respectively (mean ± SD; n = 30 plants each treatment). (d) Response of *fca-1* mutant plants to salt stress (mean ± SD; n = 40 plants each treatment). The different letters indicate a significant difference at P <0.05. Taken from: (Li et al., 2007).

Q4) "215 mutant *gi-2* (Figure 2A,B). These results support the hypothesis that only the nuclear pool of GI is competent to promote photoperiodic flowering...." This conclusion is not fully supported since proGI:GI-GFP-NES gi-2 are much earlier flowering compared to *gi-2* (Fig 2B). Why? The authors should at least quantify GI in nuclear fractions along these lines.

Although the transgenic line *GI-NES*/*gi-2* produces a recombinant GI tagged with a nuclear exclusion signal, a fraction of it (*ca*. 10%) was still detected within the nucleus, whereas the *gi-2* plant does not produce a functional GI protein at all (Kim et al., 2013b) (see figure below). Because the nuclear exclusion of GI-NES is not complete, differences in flowering time relative to the *gi-2* mutant are to be expected.

Since the notion that the nuclear pool of GI is the critical one controlling photoperiodic flowering was proposed elsewhere (Kim et al., 2013b), we have changed the conflicting sentence to read: These results are coherent with the finding that only the nuclear pool of GI was competent to promote photoperiodic flowering (Kim et al., 2013a), and suggest that the salt-induced delay of flowering, which results from a decrease in the steady-state levels of total GI protein (Kim et al., 2013a), affects primarily the cytosolic GI pool. (lines 188-192).

Figure S1. Nucleus-Cytosol-Localized GI Have Differential Roles Controlling Circadian Outputs in GI-NLS and GI-NES Transgenic

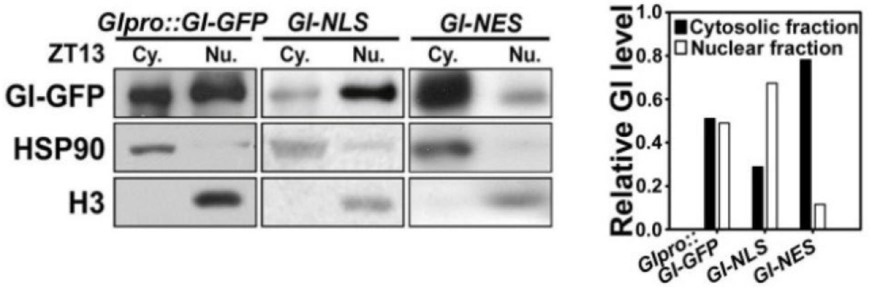

and

in

GI-

Plants. GI protein localization in transgenic GIpro::GI-GFP, GI-NLS and GI- NES plants. GI proteins were detected in 10-day-old seedlings grown under 12L/12D conditions at ZT13. Cytosol- and nucleus-enriched fractions were indicated by HSP90 and H3, respectively. Note that even though GI-NLS plants have a residual amount of cytosolic GI and GI-NES plants have a residual amount of nuclear GI, microarray analysis revealed that GI-NLS and GI-NES plants can have distinct nuclear- and cytosol-dependent gene expression patterns (Figure 1D). Taken from: (Kim et al., 2013c)

Q5) It is not clear how/ if the reduced levels of nuclear GI in sos3 mutants specifically depends on a protective role of nuclear SOS3 on GI. This should be addressed more directly. Also, if the assumption is that cytosolic GI (and de novo translated GI) undergo proteolysis under NaCl conditions, it might be expected that over time the nuclear GI pool would be gradually depleted. Longer time course experiments could verify this prediction.

The Reviewer is right, the nuclear GI pool is expected to deplete over time, which explains in molecular terms the delayed flowering time under salt stress. Please note that GI is a nuclear-cytoplasmic protein whose partition between compartments is dynamic (Kim et al., 2013b), which implies that depletion of the cytosolic pool likely draws more GI from the nuclei towards the cytosolic compartment. Here we show that, on top of that, the nuclear-localized GI is somehow protected from degradation by the interacting protein SOS3, in the absence of which GI becomes labile. The simplest explanation is that the GI-SOS3 complex prevents GI degradation, although more complex scenarios cannot be ruled out at this time. Furthermore, we have analyzed transcription of *CO* and *FT*, which is dependent on the amount of GI protein, at longer times of salinity treatments. Data presented in Supplemental Fig. S2 supports the notion that a functional SOS3 protein fosters *CO* and *FT* gene expression at longer times of exposure to salt. We explain this in the Discussion, lines 445-451.

Nuclei have proteasomes that could degrade unprotected GI inside the nucleus or, alternatively, more GI could be partition to the cytoplasm in *sos3* mutant plants and be degraded there. Moreover, we do not know the molar ratio of SOS3 vs GI inside the nucleus, which may ultimately determine how much GI protein is degraded or protected. Our data does not discriminate between these possibilities but we think that resolving this issue in that fine detail is not critical to the main conclusion and message (nuclear import of SOS3 stabilizes nuclear GI under salt stress) of this already extensive work.

Q6) The interpretation of the ChIP-qPCR in Fig7B is unclear. GI association with the CO promoter is negatively influenced by salt. However, GI accumulation in nuclei does not seem to be affected by salt. What is the reason for reduced GI occupancy at the promoter of CO? Unless the cytosolic GI pool affects the function of nuclear GI.

GI is a very large and complex protein, mainly considered to be a scaffold or even a co- chaperone (Cha et al., 2017), whose activity is modulated by an increasing number of physiological effectors. The selective binding of different partnering proteins determines GI-dependent outputs in several physiological processes. One of GI's major roles is being part of the clock, and this is regulated by the evening complex in association with ELF4. In the photoperiodic flowering pathway, GI associates with FKF1 and ZTL, which controls both GI subcellular partition and binding to the *CO* promoter. GI can also associate with TCP4 to bind to the *CO* promoter (Kubota et al., 2017). Last, the *CO* promoter itself is busily occupied by BHLHs, CDFs and a plethora of chromatin remodeling proteins that control *CO* transcription during the evening of a LD (He et al., 2020). Therefore, recruiting of GI to the *CO* promoter, which here we show is influenced by salt, could be a separate factor from its concentration in the nucleus, as the role of GI as transcriptional co-factor is regulated by a rather complex mechanism involving many other proteins. It is easy to envision that several other factors controlling GI occupancy of the *CO* promoter under saline stress will surely be at play in addition to SOS3. However, we think that these issues will require additional research that, again, goes beyond the scope this work.

Q7) Other extra points: Panels in Fig 2 read a bit weird in terms of numbering. Consider improving the arrangement to match the text. I also suggest to state the promoter used above each panel instead of referring to e.g. GI-GFP (35S-GI-GFP etc)

We have reorganized the layout of panels in Fig. 2. Thank you for the recommendation regarding the labeling of panels. However, if we noted the *35S* promoter in labels in Fig 2 we should do the same in Fig 1, Fig. 3 and Fig. 5 Some of these figures are quite 'crowded' already, and adding more characters does not seem beneficial for visual clarity, specially when the promoter used for every construct is stated in the main text, methods and the figure legends.

Q8) There are notable differences between the RT-qPCR analyses on CO transcript levels presented in FIG1 C and FIG S1 regarding NaCl treatment effects on WT and sos3-1 mutants. It is important to resolve this since the graph in FIG.1 points to alterations in CO transcript in response to salt depending on the genetic background while FIG. S1 shows minimal differences.

These experiments reflect the behavior at short- and long-term salt treatments. Please note that maximal differences in *CO* transcript abundance in Fig 1C were recorded at night (ZT20 and ZT24), with minor differences at other time points. Moreover, the experiment in Fig 1 was made with 2-week old plants and the experiment in Supplemental Fig S2 used 8-week old plants. Therefore, expression data should not be compared in terms of absolute values but genotype x treatment interactions. In this regard, the two data sets are coherent and support the notion that SOS3 is a positive effector of *CO* and *GI* transcription under salt stress.

Q9) (Figure 1A,B). These results suggest that GI promotion of flowering strictly requires a functional SOS3 protein under salinity stress but not in regular growth conditions. It would be informative to qualify this statement with appropriate statistics. From Fig 1A sos3 GI:HA plants seem to be slightly later flowering than GI:HA w/out NaCl.

For the revised manuscript we provide a new Fig 1B in which we show the statistical comparison of all means to make our conclusions easier to visualize. The data shows that GI promotion of flowering under salinity stress requires a functional SOS3 protein because only the *sos3-1* mutant had an extra-delayed flowering time under saline stress among all genotypes tested, a defect that could not be compensated by GI overexpression (both the only category 'c' in Fig 1B). Through additional research, this phenotype could be linked to the nuclear import of SOS3 and GI protein stability under salt stress.

As commented above to Reviewer #1, GI overexpression in the WT background produced early flowering under control conditions that is different (category 'd') to that of *sos1/GI*, *sos2/GI* and *sos3/GI* (category 'f'). This could be due to the capacity of GI to interact with all SOS proteins. We have shown previously that GI forms a complex with SOS2 (Kim et al., 2013a), and we report here that GI also forms a complex with SOS3. In another unpublished work (provisional acceptance in *P.N.A.S.*) we have found that GI interacts with SOS1, and this last interaction results in altered circadian clock under salt stress. How the loss of these SOS-GI interactions in the *sos1/2/3* mutants, which are retained in the WT, affect GI function in setting flowering time under regular non-saline conditions is not entirely understood and will require additional research. We believe that speculations about these more subtle differences add little to the main conclusions and fall outside the focus of this work. In this paper we intend to provide a molecular basis for the critical observation of extra-delayed flowering time in the *sos3* mutant in a saline environment, which is the most prominent difference quantified in Fig 1B.

Q10) Please state which normalization method has been used to quantify fold change in qPCRs (delta delta Ct?).

The relative expression levels was normalized by the comparative cycle threshold (ΔΔCt) method (Livak and Schmittgen, 2001). This info has been incorporated to Supplemental Methods and the legend to Figure 1.

Q11) In material and methods "Seeds were sterilized, vernalized for two days at 4ºC" change into stratified.

Changed.

<u>Reviewer #3:</u>

General comment: It happens that I have already reviewed this paper previously submitted to another journal. I was rather positive about this work and recommended it publication after some additional experiments and clarification of the few issues. Most of them have been incorporated in the current version, and I believe the paper can be published almost as it is.

Thank you for the additional effort to revise this manuscript again, and for the positive comment!

Q1) The authors showed that the palmitoylation directs SOS3 nuclear import, is it induced by salt stress?

Yes, salt stress induced palmitoylation-dependent nuclear import of SOS3. The data is in Fig 4F. Please, compare mock and salt treatments (white and blue columns) and the relative SOS3 protein abundance in cytoplasm and nuclei in these conditions.

Q2) Only one line used for phenotypic analyses is insufficient.

Only one representative line was used when the transgenic/mutant had been described elsewhere and the consistent phenotype assessed at the time of production (e.g. the GI-GFP, GI-NLS and GI-NES lines in Fig. 2) (Kim et al., 2013b). The *sos1,2,3* mutant lines expressing GI-HA (Fig 1 & 2) where obtained by genetic crossing, meaning that all shared the same transgenic event that had been previously characterized (Kim et al., 2013a). Proper references to these previously characterized materials are given in the manuscript. We believe this is the standard procedure with published biological resources. Please note that transgenic/complemented lines produced specifically for this work were selected as representative lines after comparing several independent lines representing unique transformation events (e.g. *sos3-1* complemented lines with SOS3 protein variants, see Supplemental Fig S7), and the *pSOS3:SOS3-GFP/sos3-1* line used for spinning disc confocal microscopy (see Supplemental Fig S6).

REFERENCES

Cha, J.Y., Kim, J., Kim, T.S., Zeng, Q., Wang, L., Lee, S.Y., Kim, W.Y., and Somers, D.E. (2017). GIGANTEA is a co-chaperone which facilitates maturation of ZEITLUPE in the Arabidopsis circadian clock. Nat Commun 8, 3.

He, Y., Chen, T., and Zeng, X. (2020). Genetic and Epigenetic Understanding of the Seasonal Timing of Flowering. Plant communications 1, 100008.

Kim, W.-Y., Ali, Z., Park, H.J., Park, S.J., Cha, J.-Y., Perez-Hormaeche, J., Quintero, F.J.,

Shin, G., Kim, M.R., Qiang, Z., Ning, L., Park, H.C., Lee, S.Y., Bressan, R.A., Pardo, J.M., Bohnert, H.J., and Yun, D.-J. (2013a). Release of SOS2 kinase from sequestration with GIGANTEA determines salt tolerance in Arabidopsis. Nat Commun 4, 1352.

Kim, Y., Han, S., Yeom, M., Kim, H., Lim, J., Cha, J.-Y., Kim, W.-Y., Somers, David E., Putterill, J., Nam, Hong G., and Hwang, D. (2013b). Balanced nucleocytosolic partitioning defines a spatial network to coordinate circadian physiology in plants. Developmental Cell 26, 73-85.

Kim, Y., Han, S., Yeom, M., Kim, H., Lim, J., Cha, J.Y., Kim, W.Y., Somers, D.E., Putterill, J., Nam, H.G., and Hwang, D. (2013c). Balanced nucleocytosolic partitioning defines a spatial network to coordinate circadian physiology in plants. Dev Cell 26, 73-85.

Kubota, A., Ito, S., Shim, J.S., Johnson, R.S., Song, Y.H., Breton, G., Goralogia, G.S., Kwon, M.S., Laboy Cintrón, D., Koyama, T., Ohme-Takagi, M., Pruneda-Paz, J.L., Kay, S.A., MacCoss, M.J., and Imaizumi, T. (2017). TCP4-dependent induction of CONSTANS transcription requires GIGANTEA in photoperiodic flowering in Arabidopsis. PLoS Genet 13, e1006856.

Li, K., Wang, Y., Han, C., Zhang, W., Jia, H., and Li, X. (2007). GA signaling and CO/FT regulatory module mediate salt-induced late flowering in Arabidopsis thaliana. Plant Growth Regul 53, 195-206.

Livak, K.J., and Schmittgen, T.D. (2001). Analysis of relative gene expression data using real-time quantitative PCR and the 2(-Delta Delta C(T)) Method. Methods 25, 402-408.

TPC2021-RA-00153R1 2nd Editorial decision – *accept with minor revision* Aug. 24, 2022

We have received reviews of your manuscript entitled "S-acylated and Nuclear-Localized SOS3/CBL4 Stabilizes GIGANTEA to Regulate Arabidopsis Flowering Time under Saline Stress." On the basis of the advice received, the board of reviewing editors would like to accept your manuscript for publication in The Plant Cell. This acceptance is contingent on revision based on the comments of our reviewers. In particular, please consider the following:

Please add some discussion to highlight the idea that salt stress may also impair proper GI function (or its complex formation) in the nucleus, as requested by reviewer 2. Please highlight all changes and include a detailed annotation of changes of the text, with line numbers, and noting your responses to the comments.

A note from the Editor-in-Chief: We are trying to make a concerted effort to change green/red comparisons

to green/magenta to make our figures understandable to those with color vision deficiencies. We noticed that a small number of your figures utilize red/green contrasts, so as you prepare the final version of the figures, please check the figures for red/green color use. Magenta is a good substitute for red, and a light blue substitutes for green even if the data are labeled as "RFP" or "mCherry" - readers will understand that colors can be changed, and indeed, it can be adjusted relatively quickly by using the "hue" setting in Photoshop or similar software. For example, Figure 7 may need to be corrected; you might want to check over the Supplemental Figures for similar comparisons. Please note that color changes do not need to be highlighted or tracked in the revised manuscript, but could be noted in the cover letter or response document.

-- Reviewer comments:

Reviewer #1:

The authors have resolved all my questions.

Reviewer #2:

Thanks to the authors for incorporating the suggestions made and for their clarifications. I am overall satisfied with this version of the study.

I only have one more suggestion regarding the interpretation of the ChiP result. The authors' response (below) is convincing and I suggest adding a few lines to their discussion to highlight the idea that salt stress may also impair proper GI function (or its complex formation) in the nucleus.

"Therefore, recruiting of GI to the CO promoter, which here we show is influenced by salt, could be a separate factor from its concentration in the nucleus, as the role of GI as transcriptional co-factor is regulated by a rather complex mechanism involving many other proteins. It is easy to envision that several other factors controlling GI occupancy of the CO promoter under saline stress will surely be at play in addition to SOS3. "

Reviewer #3:

The authors have addressed my comments. I do not have further questions.

TPC2021-RA-00153R2 2nd Revision received Aug. 29, 2022

Author responses:

We have incorporated to Discussion the concept that other factors controlling GI occupancy of the *CO* promoter under saline stress will be at play in addition to SOS3, as suggested by Reviewer 2 (see lines 451-466).

Fig. 6D and Supplemental Fig S10. Red arrowheads over a green fluorescence background changed to white.

Fig. 7. Green fluorescence changed to yellow, and red to magenta.

Fig 8D and Supplemental Fig S3B. Red boxes in plots changed to blue.

Fig S11B. Red columns in plot changed to magenta.

TPC2021-RA-00153R2 3rd Editorial decision – *acceptance pending* Sept. 1, 2022

We are pleased to inform you that your paper entitled "S-acylated and Nuclear-Localized SOS3/CBL4 Stabilizes GIGANTEA to Regulate Arabidopsis Flowering Time under Saline Stress" has been accepted for publication in The Plant Cell, pending a final editorial review by a science editor.

Final acceptance from Science Editor **Sept. 16, 2022**
